# CT-Based Simulation of Left Ventricular Hemodynamics: A Pilot Study in Mitral Regurgitation and Left Ventricle Aneurysm Patients

**DOI:** 10.3389/fcvm.2022.828556

**Published:** 2022-03-22

**Authors:** Lukas Obermeier, Katharina Vellguth, Adriano Schlief, Lennart Tautz, Jan Bruening, Christoph Knosalla, Titus Kuehne, Natalia Solowjowa, Leonid Goubergrits

**Affiliations:** ^1^Institute of Computer-Assisted Cardiovascular Medicine, Charité - Universitätsmedizin Berlin, Berlin, Germany; ^2^Fraunhofer Institute for Digital Medicine MEVIS, Bremen, Germany; ^3^Department of Cardiothoracic and Vascular Surgery, German Heart Center Berlin, Berlin, Germany; ^4^DZHK (German Centre for Cardiovascular Research), Partner Site Berlin, Berlin, Germany; ^5^Charité - Universitätsmedizin Berlin, Corporate Member of Freie Universität Berlin, Humboldt - Universität zu Berlin and Berlin Institute of Health, Berlin, Germany; ^6^Department of Congenital Heart Disease, German Heart Center Berlin, Berlin, Germany; ^7^Einstein Center Digital Future, Berlin, Germany

**Keywords:** cardiac computed tomography, intraventricular hemodynamics, image-based modeling, left ventricle aneurysm, mitral regurgitation, fluid-structure interaction, computational fluid dynamics

## Abstract

**Background:**

Cardiac CT (CCT) is well suited for a detailed analysis of heart structures due to its high spatial resolution, but in contrast to MRI and echocardiography, CCT does not allow an assessment of intracardiac flow. Computational fluid dynamics (CFD) can complement this shortcoming. It enables the computation of hemodynamics at a high spatio-temporal resolution based on medical images. The aim of this proposed study is to establish a CCT-based CFD methodology for the analysis of left ventricle (LV) hemodynamics and to assess the usability of the computational framework for clinical practice.

**Materials and Methods:**

The methodology is demonstrated by means of four cases selected from a cohort of 125 multiphase CCT examinations of heart failure patients. These cases represent subcohorts of patients with and without LV aneurysm and with severe and no mitral regurgitation (MR). All selected LVs are dilated and characterized by a reduced ejection fraction (EF). End-diastolic and end-systolic image data was used to reconstruct LV geometries with 2D valves as well as the ventricular movement. The intraventricular hemodynamics were computed with a prescribed-motion CFD approach and evaluated in terms of large-scale flow patterns, energetic behavior, and intraventricular washout.

**Results:**

In the MR patients, a disrupted E-wave jet, a fragmentary diastolic vortex formation and an increased specific energy dissipation in systole are observed. In all cases, regions with an impaired washout are visible. The results furthermore indicate that considering several cycles might provide a more detailed view of the washout process. The pre-processing times and computational expenses are in reach of clinical feasibility.

**Conclusion:**

The proposed CCT-based CFD method allows to compute patient-specific intraventricular hemodynamics and thus complements the informative value of CCT. The method can be applied to any CCT data of common quality and represents a fair balance between model accuracy and overall expenses. With further model enhancements, the computational framework has the potential to be embedded in clinical routine workflows, to support clinical decision making and treatment planning.

## 1. Introduction

Cardiac CT (CCT) is a preferred imaging modality for the treatment of various cardiovascular diseases and is for example used in the investigation of coronary artery disease, for the measurement of myocardial perfusion, or in the planning of interventions in valvular heart diseases (1, 2). For surgical or transcatheter aortic valve (AV) implantation, CCT is currently state of the art imaging technique (3–5). Due to a superior spatial resolution (0.5–0.625 mm), if compared to MRI (1–2 mm), or echocardiography (0.5–2 mm) (6), CCT allows for assessment and quantification of anatomical structures necessary for decision making and treatment planning (5). However, in clinical practice, intracardiac blood flow analysis is based on MRI or Doppler echocardiography, as CCT cannot capture flow quantities like blood velocity and pressure. Recently, Lantz et al. (7) demonstrated the potential of coupling CCT with computational fluid dynamics (CFD) for so-called 4D flow CCT that allows the computation of intraventricular hemodynamics based on CCT. Despite being more time-consuming in post-processing and in general, less investigated and established, the methodology potentially enables investigations at higher spatial resolutions as well as a reduction of acquisition time, and may thus be a promising alternative to 4D flow MRI (8).

Intraventricular blood flow behavior is believed to potentially serve as an early predictor for the manifestation of diseases (9) and is used to investigate pathological states (10, 11) or the success assessment of interventional procedures for heart diseases (12). It is furthermore used for the analysis of post-operative states like intraventricular flow with prosthetic mitral valves (MVs) (13) or the influence of annuloplasty rings (14). The *in vivo* analysis of intraventricular blood flow *via* medical images includes, for example, the quantification of blood flow kinetic energy (15, 16), the characterization of the LV hemodynamics *via* the analysis of large-scale flow patterns (17–22), intraventricular pressure gradient analysis (23) or diastolic vortex analysis in terms of formation time in early diastole (10), vortex strength (11) or rotational vortex direction (16). Techniques and quantities for intracardiac flow analysis were recently overviewed by Mele et al. (24).

Due to advancements in medical image acquisition and numerical modeling, as well as growing computational resources, the use of image-based patient-specific models for the analysis of intraventricular hemodynamics increased in recent years. Image-based CFD approaches are for example used to investigate intraventricular flow patterns and diastolic vortex formation (25–28) as well as loss of kinetic energy and pressure gradients in LVs (29). In contrast to medical imaging techniques, CFD allows to analyze wall shear stress at high spatio-temporal resolutions (30) and to model post-operative states, e.g., after MV treatment (12, 31) or surgical ventricular restoration (32). CFD furthermore enables the creation of synthetic cases, for example, LVs *via* statistical shape models (33) or different incorporated MV shapes in mitral regurgitation (MR) (34). Proposed modeling approaches vary significantly regarding complexity, ranging from 0D Lumped Element Models, where the cardiovascular system is described as an electro-mechanical circuit (35, 36), *via* image-based CFD approaches where the LV motion is prescribed as boundary condition (BC) (25, 26), to complex models coupling electro-, structural and fluid mechanics, as for example used in the Living Heart Project (37, 38). Doost et al. (39) gave a detailed review of heart blood flow simulation approaches and attributed the image-based, prescribed-motion CFD models the greatest popularity due to lower computational costs and their convenience in comparison to coupled fluid-structure-interaction (FSI) frameworks. An overview of different modeling approaches of FSI and a review on the current state of the art FSI modeling in cardiovascular medicine was recently given by Quarteroni et al. (40) and Hirschhorn et al. (41).

The balancing between model accuracy and complexity marks a central trade-off in the CFD-based investigation of LV hemodynamics. Modeling approaches need to incorporate the most important features to accurately reproduce the intraventricular hemodynamics. At the same time, data requirements, required working hours, computational expenses, and model uncertainties need to be minimized to enable a usability in the clinical routine. Finding an adequate compromise is the subject of this study. We propose a moderate complexity computational framework to compute LV hemodynamics by using CCT image-based, prescribed-motion CFD that requires pre-processing times and computational expenses that are within reach of clinical feasibility. The workflow is largely automated and applicable to arbitrary CCT data of common quality. The technical feasibility is demonstrated by means of four cases, each representing a subcohort with a respective pathological configuration: patients with and without LV aneurysm as well as with severe and no MR.

## 2. Materials and Methods

**Figure 1:**
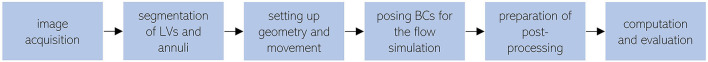
Illustration of the central workflow of the computational framework.

The central workflow of the computational framework is illustrated in Figure 1. Following the image acquisition *via* CCT, the image data is used to segment end-diastolic and end-systolic LV geometries as well as both annuli. In the next step, the segmented data are used to set up LV geometries with 2D valves and to derive the ventricular movement. For the intracardiac flow simulations, adequate physics models and BCs are furthermore posed. As the last step, valuable hemodynamic markers for the analysis of intraventricular flow features are selected before each of the four patient-specific cases is computed and evaluated. The individual steps are addressed in detail in the following.

### 2.1. Study Cohort

A cohort of heart failure patients after myocardial infarction (*n* = 125, mean age of 60.6 ± 10.0 years, 16.8 % women) was retrospectively analyzed. Data were collected in the German Heart Center Berlin between November 2005 and January 2016. The patients are grouped into two cohorts: patients without (Cohort I) and with (Cohort II) anterior LV aneurysm. The LV aneurysm was diagnosed by echocardiography and confirmed by CCT. The MR grade was defined echocardiographically. For each cohort, one case without MR and one with severe MR (grade III) were selected. The different cases are denominated *via* M{MR: 0 = no MR; 1, 2, 3 = grade I, II, III}A{aneurysm: 0 = no; 1 = yes}, resulting in the four cases: M3A1, M3A0, M0A1, and M0A0. The hemodynamics of all cases are investigated, each being representative for its subcohort. The four LVs were chosen such that the end-diastolic volumes (226 to 256 ml) and the reduced ejection fractions (EFs) (23 to 29 %) are comparable. Table 1 summarizes the clinical and demographic data of the two cohorts and the four representative cases. Statistical analysis was performed with SPSS (version 27, IBM, Armonk, NY, USA), whereat a *p*-value below 0.05 was considered statistically significant.

**Table 1:** Clinical and demographic data of the two study cohorts and the four representative cases.

**Parameters**	**Cohort I (A0)**	**Cohort II (A1)**	**M3A1**	**M3A0**	**M0A1**	**M0A0**
Nr. of cases	72	53	-	-	-	-
LV aneurysm	no	yes	yes	no	yes	no
Age (years)	60 [54 - 69]	59 [54 - 68]	60	60	49	62
Sex (m/w)	62/10	42/11	m	m	m	m
BSA (m^2^)	1.98 ± 0.19	1.91 ± 0.23	2.18	2.02	1.76	2.36
MR (grade)	I [I - II/III]	I [I - II]	III	III	-	-
NYHA class	III [III - III]	III [III - III]	III	IV	III	III

*Cohort p-values range from 0.143 to 0.676 and are not considered significant. If a parameter is not normally distributed in either cohort, values are shown as median [interquartile range (IQR)] for both cohorts, whereas values are shown as mean ± standard deviation (SD) otherwise. Normality was proved by the Shapiro-Wilk test. The BSA was calculated according to the Dubois formula (42). LV, left ventricle; BSA: body surface area; MR, mitral regurgitation; NYHA, New York Heart Association*.

### 2.2. Computed Tomography

Cardiac CT examinations were performed following injection of intravenous contrast agent. The scans were performed in a spiral modus with retrospective electrocardiogram-gating, using a dual-source multi-slice spiral CT scanner (Somatom Definition Flash, Siemens Healthcare GmbH, Erlangen, Germany). All scans were conducted at a tube voltage of 100 kV and an individually adapted tube current, using the scanners exposure control software. A multiphase data set of the whole heart cycle was reconstructed that allows identification of the diastolic phase with the largest left ventricular end-diastolic volume (LVEDV) and the systolic phase with the smallest left ventricular end-systolic volume (LVESV). All images were reconstructed using a standard soft-tissue convolution kernel and a dedicated noise reduction software. The spatial and temporal resolution of the CT images varied. Spatial resolutions of (0.39–0.648 mm) x (0.39–0.648 mm) in-plane resolution and (0.5–1.85 mm) slice thickness were used for segmentation. The temporal resolution ranged from 70 to 140 ms depending on the patient's heart rate. One heart cycle was always resolved by 10 phases.

### 2.3. Segmentation

**Figure 2:**
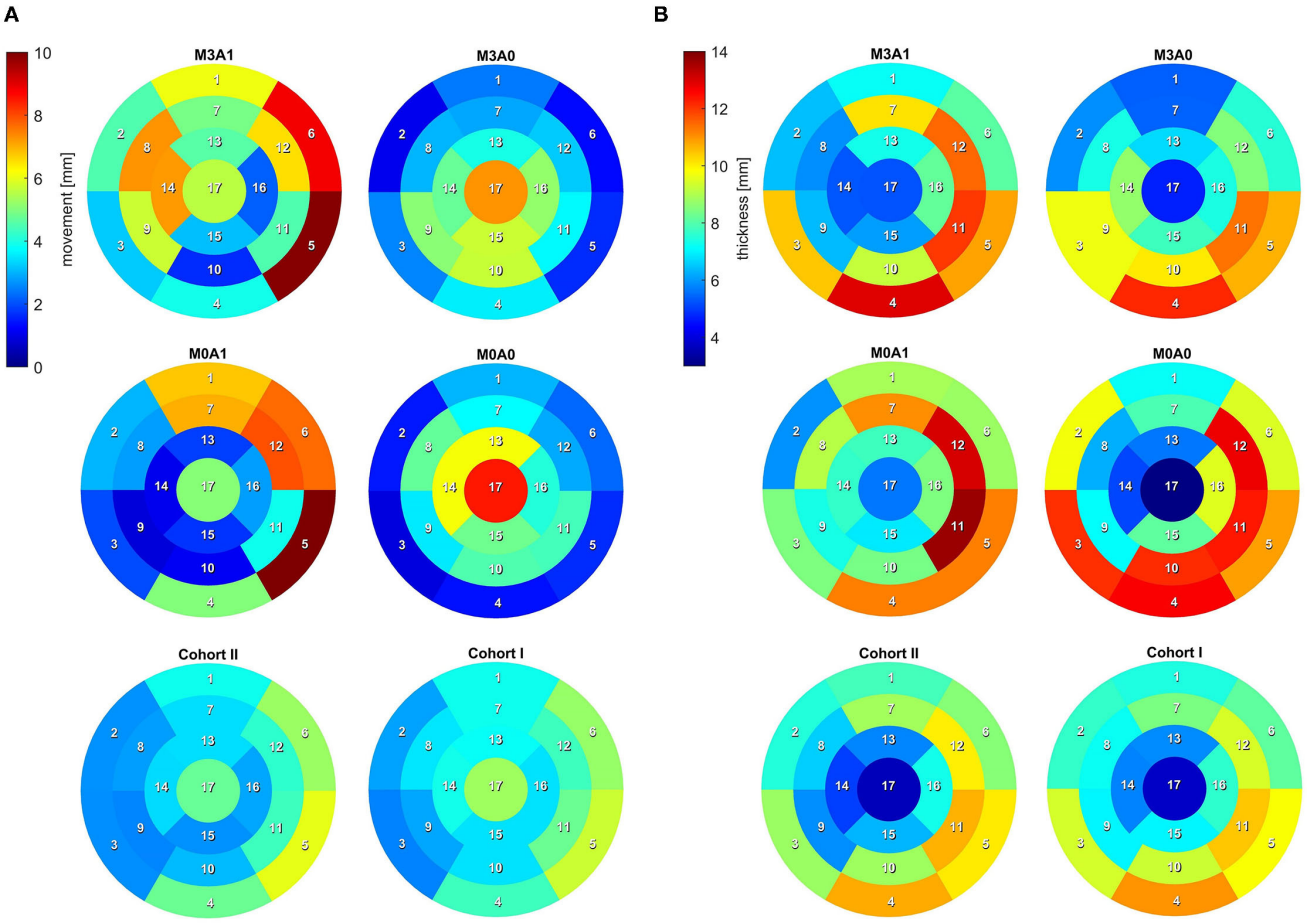
17-segment representation of **(A)** the myocardial wall movement (averaged wall distance from LVEDV to LVESV per segment) and **(B)** the myocardial wall thickness (segmented for the LVEDV). Data are shown averaged for Cohort I and II as well as for the four representative cases.

To analyze the LV anatomy as well as to prepare geometric and dynamic boundary conditions for the flow simulations, the LVs were segmented. The segmentations were created using a prototypical software developed earlier on the basis of the MeVisLab platform (43). A detailed description of the segmentation procedure can be found in Tautz et al. (44). In a first step, segmentations of the end-diastolic and end-systolic LVs were done automatically. The automated procedure is based on an adaptive 3D region growing approach, detection of the axial view of the ascending aorta (AO), and taking into account contextual information of the heart topology. Nevertheless, a manual correction was required to fix problems associated with artifacts due to implanted metal parts or uneven distribution of contrast agents. The segmentation of the end-diastolic myocardial wall was done manually based on the previously segmented LV. Subsequently, the mitral and aortic annuli were segmented manually by defining 36 landmarks in altogether 18 2D image planes, rotated around the estimated valvular axis. These landmarks were used to interpolate the aortic annulus onto an ellipse and the mitral annulus onto a cardioid. As the last step, three landmarks were set manually to define the right coronary artery ostium as well as the tips of the anterior and posterior papillary muscles. The right coronary artery was used for the registration of the end-diastolic and end-systolic geometry, necessary to implement 17-segment visualizations of the LV. The 17-segment visualizations of the myocardial wall movement and wall thickness are shown in Figures 2A,B for both cohorts and the four representative cases. For details on the 17-segment visualizations, the reader is referred to Cerqueira et al. (45). The segmented lumina were saved as DICOM files and used to generate triangulated surfaces required for the computation of hemodynamics and the measurement of selected geometric parameters including LVEDV and LVESV, LV sphericity index, stroke volume (SV), EF, and the areas of both annuli. It is to note, that LVEDV, LVESV, and SV slightly increase when reconstructing the LV annuli area, while the EF is kept constant (see section 2.4). The sphericity index is calculated based on the LV volume and the LV long axis length according to the empiric formula in Equation (1) (46).


(1) $$\begin{array}{l} \text{SI}=\frac{6\cdot \text{volume}}{{\left(\text{long axis}\right)}^{3}\cdot \pi } \end{array}$$

**Figure 3:**
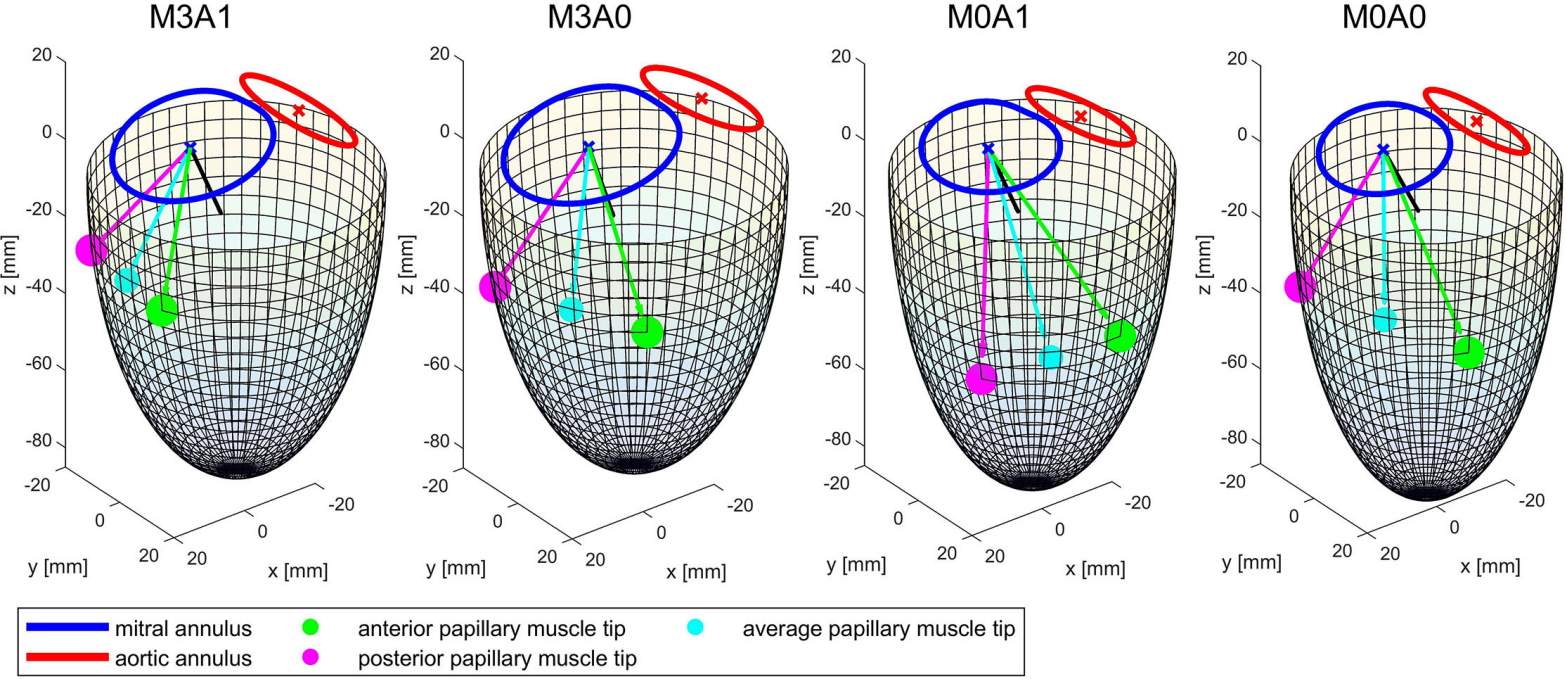
Wireframe visualizations of the four representative cases at the end of systole. The papillary muscle tips are projected on the surface of the half-ellipsoid. The black line shows the vector normal on the mitral annulus plane. The cyan line and dot mark the average of the vectors connecting the papillary muscle tips with the center of the mitral annulus. It represents the direction of an averaged force of the chordae tendineae acting on the MV leaflets.

Table 2 summarizes the geometric parameters of both cohorts and the four representative cases. Figure 3 displays a 3D wire-frame representation of the four investigated cases for visual comparison of the LV sphericity, annuli sizes, and positioning, as well as the location of the papillary muscle tips. Therein, the LVs are simplified by a half-ellipsoid of the case-respective LV volume and sphericity index.

**Table 2:** Geometric parameters of the two investigated cohorts and the four representative cases.

**Parameters**	**Cohort I (A0)**	**Cohort II (A1)**	**M3A1**	**M3A0**	**M0A1**	**M0A0**
LVEDV (ml)	287 [228 - 349]	284 [233 - 356]	256	248	251	226
LVESV (ml)	208 [167 - 269]	218 [177 - 285]	181	191	181	170
SV (ml)	71 [59 - 84]	66 [54 - 89]	75	57	70	56
EF (%)	24.6 [20.6 - 29.7]	22.8 [19.2 - 27.7]	29.3	23.1	27.7	24.7
EDSI	0.83 ± 0.18	0.83 ± 0.19	0.88	0.76	0.67	0.56
ESSI	0.73 [0.62 - 0.88]	0.77 [0.62 - 0.91]	0.78	0.81	0.64	0.55
MWT (mm)	8.22 [7.21 - 9.28]	8.11 [7.02 - 8.55]	8.57	8.53	9.16	9.27
WM (mm)	3.72 [3.24 - 4.55]	3.63 [2.76 - 4.37]	5.22	3.59	3.95	3.50
ED AAA (cm^2^)	5.80 ± 0.98	5.56 ± 0.96	6.30	6.45	5.30	5.32
ED MAA (cm^2^)	10.73 ± 2.23	10.63 ± 2.64	12.13	11.91	10.20	8.28

*Cohort p-values range from 0.124 to 0.834 and are not considered significant. If a parameter is not normally distributed in either cohort, values are shown as median [IQR] for both cohorts, whereas values are shown as mean ± SD otherwise. Normality was proved by the Shapiro-Wilk test. LVEDV, left ventricular end-diastolic volume; LVESV, left ventricular end-systolic volume; SV, stroke volume; EF, ejection fraction; EDSI, end-diastolic sphericity index; ESSI, end-systolic sphericity index; MWT, mean myocardial wall thickness; WM, mean wall movement; ED AAA, end-diastolic aortic annulus area; ED MAA, end-diastolic mitral annulus area*.

### 2.4. Modeling Geometry and Motion

**Figure 4:**
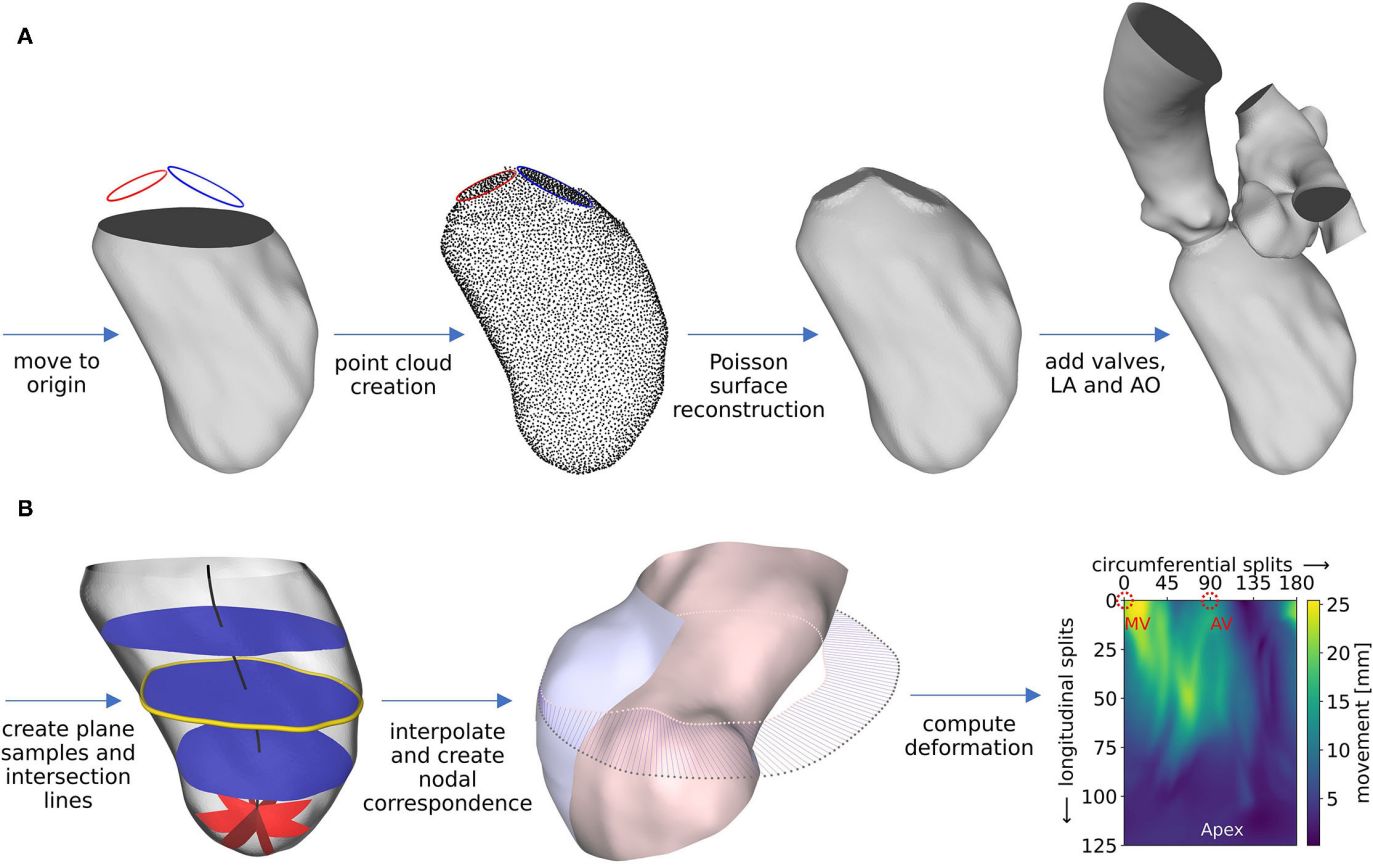
Schematic workflow representation for pre-processing of geometry **(A)** and motion **(B)**, exemplified for case M0A1. Details about the workflow are described in the text.

The segmentations are used to create well-defined surface meshes of the LVs and to derive the ventricular movement. For the sake of generalization that enables automation, the LVs are aligned *via* the MV centers. End-diastolic and end-systolic geometries are cut 1 cm below the most apical points of the AV and MV in the end-diastolic state and are slightly smoothed, preserving the LV volumes. The boundary points of that cutting plane as well as the segmented annuli are used to reconstruct a well-defined LV surface mesh of the end-diastolic geometry as follows and as schematically shown in Figure 4A. First, local coordinate axes are placed into the point clouds of the LV boundary points and both annuli *via* a best-fit plane for point clouds (47). All data is moved to the global Euclidean coordinate system, such that the LV boundary points are centered in the global origin, aligned in the *x*-*y* plane, and the vector connecting the aortic annulus center to the mitral annulus center points in the positive *x*-direction. Subsequently, points are fitted in between the annuli and the LV boundary points using a cubic polynomial function. A Poisson surface reconstruction algorithm (48) available in the open-source software Meshlab (version 2021.05, ISTI-CNR, Pisa/Rome, Italy) is applied onto the resulting point cloud to retrieve a 3D triangulation of the entire LV with interpolated annuli. To obtain well-defined 2D planes for valve modeling, the annuli are stamped into the LV. Available non-case-specific high-quality segmentations of an LA and an AO were adapted to fit the size and shape of the annuli and are attached at the respective annulus.

The ventricular motion is derived from the cut end-diastolic and end-systolic geometries (Figure 4B). With two available states, a deformation field **d**(**x**) constant in time *t* and dependent on the position **x** = (*x, y, z*) in Euclidean coordinates, can be derived. It is scaled by a time-dependent factor α(*t*) to obtain a grid velocity vector **v**(**x**, *t*) that allows the ventricular volume to follow any specified volume curve at a given EF (Equation 2).


(2) $$\begin{array}{l} \text{v}\left(\text{x},t\right)=\alpha \left(t\right)\cdot \text{d}\left(\text{x}\right) \end{array}$$

The deformation field **d**(**x**) is computed as follows. During geometry creation, the cut LVs were positioned in the global origin with the negative *z*-axis pointing toward the apex. From the highest *z*-coordinate to 1 cm above the lowest *z*-coordinate, 100 equidistantly distributed planes, being parallel to the *x*-*y* plane, are placed into the LV (as shown in Figure 4B, left, blue planes). The center of each plane is calculated, resulting in a centerline (Figure 4B, left, black line). Subsequently, the plane-LV intersection lines (Figure 4B, left, yellow line) are interpolated onto 180 points, being equidistantly distributed every 2° in azimuthal direction. Likewise, spherical plane cuts are placed in the apex region of the LV every 2° in the azimuthal direction (shown in Figure 4B, left, red planes). Again, at the intersection line of each plane with the LV, 25 points are interpolated, being equidistantly distributed in polar angle direction. The LV results to be resampled on a structured grid at 180 points in circumferential direction and 125 points in longitudinal direction, leading to a total of 22,500 sampling points at clearly defined positions. This procedure is applied to the end-diastolic and the end-systolic geometry. Figure 4B, mid, shows the LV in the end-systolic state, displayed in a half-section without the annuli region for visualization purposes (inner surface: pink, outer surface: blue). The pink dots mark the interpolated points on an exemplary plane in mid region of the LV that lie on the end-systolic surface. The gray dots mark the corresponding points in the end-diastolic state of the LV. Under the simplifying assumptions of a homogeneous longitudinal deformation and negligence of the torsional LV motion, a point correspondence between the geometries can be deduced by assigning the respective points (as shown in Figure 4B, mid, blue lines). Thereby, the deformation field of the cut LVs can be computed (Figure 4B, right). It is smoothed making use of a 3x3 Gaussian convolution kernel. The annuli, LA, and AO are simplified to be stiff. The region between the annuli and the LV boundary points is set to move in response to the deformation field at the annuli and the cut LV parts, as calculated by the STAR-CCM+ (version 2020.1, Siemens PLM Software, Plano, TX, USA) internal cubic BSpline morpher.

As the last step, the scaling factor α(*t*) is derived. It is based on a multiplicative approach and realized by forcing the volume flux of the simulation to follow the volume flux BC and results in Equation (3).


(3) $$\begin{array}{l} \alpha \left(t\right)=\frac{V\left(t+\Delta t\right)-V\left(t\right)}{\Delta t\xi \left(V^{sim}\left(t\right),\Delta t,\text{d}\left(\text{x}\right)\right)} \end{array}$$

where *t* is time, *V* is the relative volume as specified in the BC, *V*^*sim*^(*t*) is the relative volume of the simulated LV, and ξ(*V*^*sim*^(*t*), Δ*t*, **d**(**x**)) is the differential relative volume flux that a deformation along **d**(**x**) over Δ*t* at a specific volume *V*^*sim*^(*t*) induces. ξ(*V*^*sim*^(*t*), Δ*t*, **d**(**x**)) can be obtained by computing the deformation once for α(*t*) = 1 and equating ξ(*V*^*sim*^(*t*), Δ*t*, **d**(**x**)) to the resulting volume flux ${\dot{V}}^{sim}\left(t\right)$ (Equation 4).


(4) $$\begin{array}{l} \xi \left(V^{sim}\left(t\right),\Delta t,\text{d}\left(\text{x}\right)\right)={\dot{V}}^{sim}\left(t\right) \end{array}$$

Having obtained **d**(**x**) and α(*t*), the grid velocity vector (Equation 2) can be computed.

### 2.5. Governing Equations

An arbitrary Lagrangian-Eulerian method available within the STAR-CCM+ software package is utilized as discretization method. The governing equations consist of the 3*D* incompressible Navier-Stokes equations with moving mesh for unsteady flow. No body-forces and source terms are added. Therefore, the governing equations for mass (Equation 5) and momentum (Equation 6) conservation in tensor notation read as follows (49).


(5) $$\begin{array}{l} \frac{\partial }{\partial t}\int_{V}\rho \text{d}V+\int_{\Omega }\rho \left(\text{v}-{\text{v}}_{g}\right)\cdot \text{n}\text{d}\Omega =0 \end{array}$$

(6) $$\begin{array}{l} \frac{\partial }{\partial t}\int_{V}\rho \text{v}\text{d}V+\int_{\Omega }\left[\rho \text{v}\left(\text{v}-{\text{v}}_{g}\right)+p\text{I}-\text{T}\right]\cdot \text{n}\text{d}\Omega =0 \end{array}$$

Therein, *t* is time, *V* is the control volume, Ω is the boundary of the control volume, **n** is the outwardly directed vector normal to *dΩ*, ρ is density, **v** is velocity, **v**_*g*_ is the grid velocity, *p* is pressure, **T** is the viscous stress tensor, and **I** is the unit tensor of second order. The viscous stress tensor **T** for an incompressible Newtonian fluid with μ being the dynamic viscosity is shown in Equation (7).


(7) $$\begin{array}{l} \text{T}=\mu \left(\nabla \text{v}+{\left(\nabla \text{v}\right)}^{T}\right) \end{array}$$

Blood is modeled as Newtonian fluid with a density of 1,050 kgm^−3^ and a dynamic viscosity of 0.0035 Pas. The Star-CCM+ internal mesher is used to create a polyhedral volume mesh at a base size of 1 mm with a refinement to 0.25 mm in the valve region. Detailed information about the mesh can be found in the Supplementary Material.

### 2.6. Boundary Conditions and Valve Modeling

**Figure 5:**
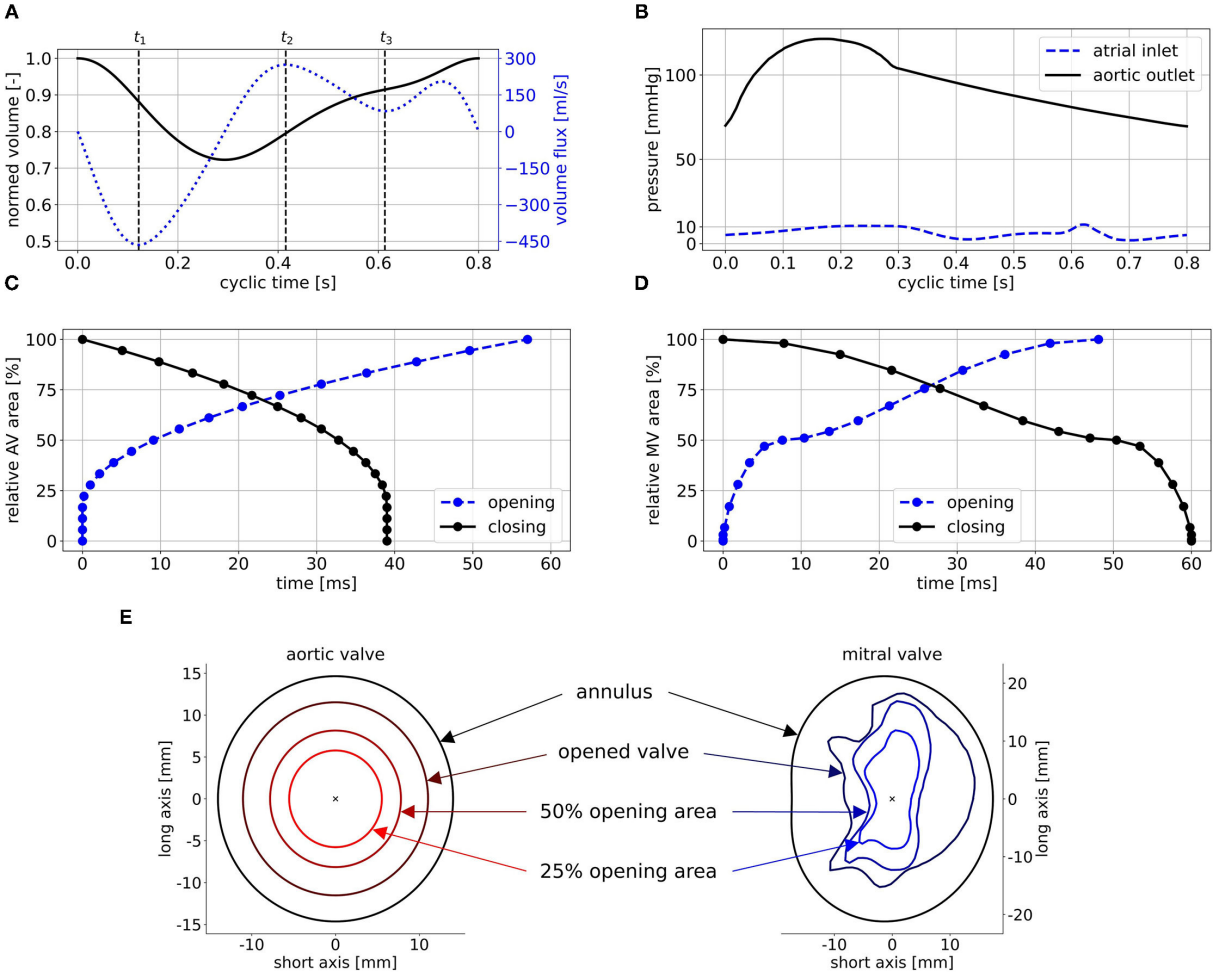
Posed BCs exemplified for case M0A1: **(A)** shows the normed volume curve and the volume flux, **(B)** the physiological pressure BCs, **(C)** and **(D)** the opening and closing times of AV and MV, and **(E)** some intermediate valve shapes of the AV (left) and the MV (right) used to realize opening and closing. Time points *t*_1_, *t*_2_, and *t*_3_ in **(A)** mark peak systole, peak E-wave, and diastasis.

The ventricular motion is prescribed as BC at the walls. No-slip BCs are furthermore posed at the walls in terms of the relative movement of the fluid to the wall. For the non-MR cases, physiological pressure BCs are extracted from circadapt (35) and posed at the aortic outlet and the atrial inlet (Figure 5B). In the MR cases, a mass flux BC is applied at the aortic outlet instead of the pressure BC. It is set to zero in diastole and to the respective proportion of the stroke volume that does not regurgitate, in systole. The regurgitation fraction is calculated based on the MR grade by correlating MR grades of I, II, and III to regurgitation fractions of 15, 30, and 50% (50). To enable comparability, the same pressure BCs and the same qualitative volume curve are utilized for all cases. The volume curve (Figure 5A) was obtained from a representative cohort by mapping all individual volume curves onto the same cyclic time, computing the first five Fourier coefficients, and averaging these. The resulting curve is then scaled onto a cyclic time of 0.8 s (75 bpm) and the case-specific EF.

The valves are implemented in a 2D-planar modeling approach (25, 51). Therein, the valve is embodied as a 2D plane, incorporating the projected orifice area of the respective valve. The valve planes are modeled as porous baffle interface, where passing fluid experiences a pressure drop according to Darcy's law (49). Advantages of this 2D-planar model are the comparably easy implementation as well as low computational costs while being able to reproduce large-scale intraventricular flow patterns (51). For the MV, a projected orifice area from the work of Schenkel et al. (25) along with its halfway opened state for opening and closing is depicted. For the AV, the orifice is assumed to be elliptical, as the aortic flow is not of particular interest in this work. Subsequently, 19 intermediate valve shapes are placed in both valve planes (Figure 5E). For the AV, they are elliptical with the same aspect-ratios and rising areas. For the MV, they are interpolated *via* a quadratic sine function between the halfway opened and the final projected orifices. By varying the pressure drop at these intermediate valve shapes, valve opening and closing as well as the general valve function is mimicked. Common areas of the MV orifice range between 4.0 and 6.0 cm^2^ (52). As all the considered LVs are dilated, a projected orifice area of 5.65 cm^2^ for the MV is chosen. This value was obtained by measuring the projected orifice area of 10 segmented MVs of patients with MR and taking the median value. For the AV, an orifice area of 4 cm^2^ is chosen, which lies at the upper end of the common range (53). The time intervals of AV opening and closing are set to 57 ms, and 39 ms, respectively, based on measured valve characteristics (54). For the MV, data availability concerning opening and closing times based on medical images is rather poor. Mao et al. (55) measured 50 ms for opening and 94 ms for closing *via* M-mode echo for one patient. In other investigations, porcine MVs (56, 57) or MVs in the ovine heart (58) were regarded. The authors reported opening times between 50 to 100 ms and closing times ranging from 50 to 150 ms. We visually inspected three MRI data sets of our own. Therein, the opening times ranged from 40 to 60 ms and the closing times from 50 to 80 ms at heart rates of 60 to 75 bpm and a temporal resolution of 40 frames per cycle. An opening time of 48 ms and a closing time of 60 ms lead to reasonable valve velocities and transvalvular pressure drops and were thus chosen. The resulting relative areas during valve opening and closing are shown in Figures 5C,D.

### 2.7. Post-processing of the LV Hemodynamics

Beside the large-scale flow patterns and the common clinical measures as transvalvular pressure differences, valve velocities, and E/A ratio, the focus is laid on the intraventricular washout and energetic efficiency. In the first cycle, a passive scalar (passive tracer that does not influence the fluid motion) is placed in the LA fluid to track the path of blood that flows into the LV in the first cycle. Therewith, a contrast agent washout can be mimicked. For the passive scalar, only convective transport is considered. To extrapolate the washout, an exponential function is used (Equation 8).


(8) $$\begin{array}{l} f\left(t\right)=e^{-B\cdot t} \end{array}$$

Therein, *t* is time and *B* is the coefficient quantifying the washout. In terms of energetic efficiency, the cycle-averaged kinetic energy is regarded. To get a hold of the energetic loss, the dissipation function Φ is used. It is defined as the double-dot product of the stress tensor **σ** and the gradient of the velocity vector ∇**v** (30, 59) as shown in Equation 9.


(9) $$\begin{array}{l} \Phi =\sigma \cdot \cdot \nabla \text{v} \end{array}$$

To visualize vortex structures, isosurfaces of the Q-criterion are used. The Q-criterion is computed *via* the second invariant of the velocity gradient tensor (60).

## 3. Results

For an experienced user, pre-processing from segmentation to a ready to process setup takes 6 - 8 h, depending on the image quality and necessity for manual rework. Computing the deformation requires 2 - 12 h, depending on the desired accuracy. By experience, a spatio-temporal resolution resulting in a 4 h computation is sufficient. The blood flow computations took 25 to 30 h per cycle on 4 nodes at 40 cores (Intel Skylake 6148) at 2.3 GHz on the Emmy system of the North-German Supercomputing Alliance. Concerning the accuracy of the deformation, the mean difference between the imposed and the computed volume curve is below 0.02 ml for all cases. The mean distances between the segmented and the computed end-systolic geometries of the four cases (when creating a nodal correspondence as described in section 2.4) are between 0.14 and 0.52 mm. The mesh quality shows no degeneration, and a mesh independence study was performed (see Supplementary Material). As the fluid is initialized in an unphysiological resting state, five cycles were computed in advance to receive a swung in the state. The consecutive eight cycles were then used for the analysis of large-scale flow patterns, energetic performance, and washout.

### 3.1. Large-Scale Flow Patterns

The intraventricular large-scale flow patterns are qualitatively evaluated by means of streamlines, first cycle diastolic inflowing blood (FCDIB), and Q-criterion. Latter two are provided in video format in the Supplementary Material (videos M3A1-FCDIB, M3A0-FCDIB, M0A1-FCDIB, M0A0-FCDIB, M3A1-Q-criterion, M3A0-Q-criterion, M0A1-Q-criterion, and M0A0-Q-criterion). Quantitative valve measures are displayed in Table 3.

**Table 3:** Quantitative valve measures of the four representative cases averaged over cycles one to eight.

**Parameters**	**M3A1**	**M3A0**	**M0A1**	**M0A0**
v MV peak E-wave (m/s)	0.75 ± 0.10	0.63 ± 0.04	0.61 ± 0.01	0.53 ± 0.01
v MV peak A-wave (m/s)	0.44 ± 0.02	0.36 ± 0.03	0.44 ± 0.01	0.35 ± 0.01
v MV peak sys (m/s)	4.58 ± 0.01	4.46 ± 0.01	-	-
v AV peak sys (m/s)	0.70 ± 0.03	0.56 ± 0.02	1.38 ± 0.04	1.10 ± 0.03
mean E/A ratio (-)	1.72 ± 0.28	1.75 ± 0.16	1.39 ± 0.04	1.49 ± 0.04
Δp MV peak E-wave (mmHg)	0.69 ± 0.25	0.40 ± 0.28	0.67 ± 0.01	0.58 ± 0.01
Δp MV peak sys (mmHg)	77.96 ± 0.56	72.96 ± 0.36	-	-
Δp AV peak sys (mmHg)	0.93 ± 0.04	0.68 ± 0.02	3.91 ± 0.13	2.59 ± 0.01
Δp_*B*_ MV peak E-wave (mmHg)	2.30 ± 0.58	1.61 ± 0.19	1.48 ± 0.03	1.11 ± 0.02
Δp_*B*_ MV peak sys (mmHg)	84.06 ± 0.35	79.56 ± 0.18	-	-
Δp_*B*_ AV peak sys (mmHg)	1.95 ± 0.18	1.25 ± 0.07	7.66 ± 0.42	4.85 ± 0.28

*Values are given in mean ± SD. The valve velocities are measured 5 mm downstream of the respective valve center. The transvalvular pressure difference is calculated via the static pressure of two point probes positioned 5 mm upstream and downstream of the respective valve center. MV, mitral valve; AV, aortic valve; Δp_B_, pressure loss calculated from Bernoulli via 4v^2^ with the respective valve velocity as specified in this table*.

**Figure 6:**
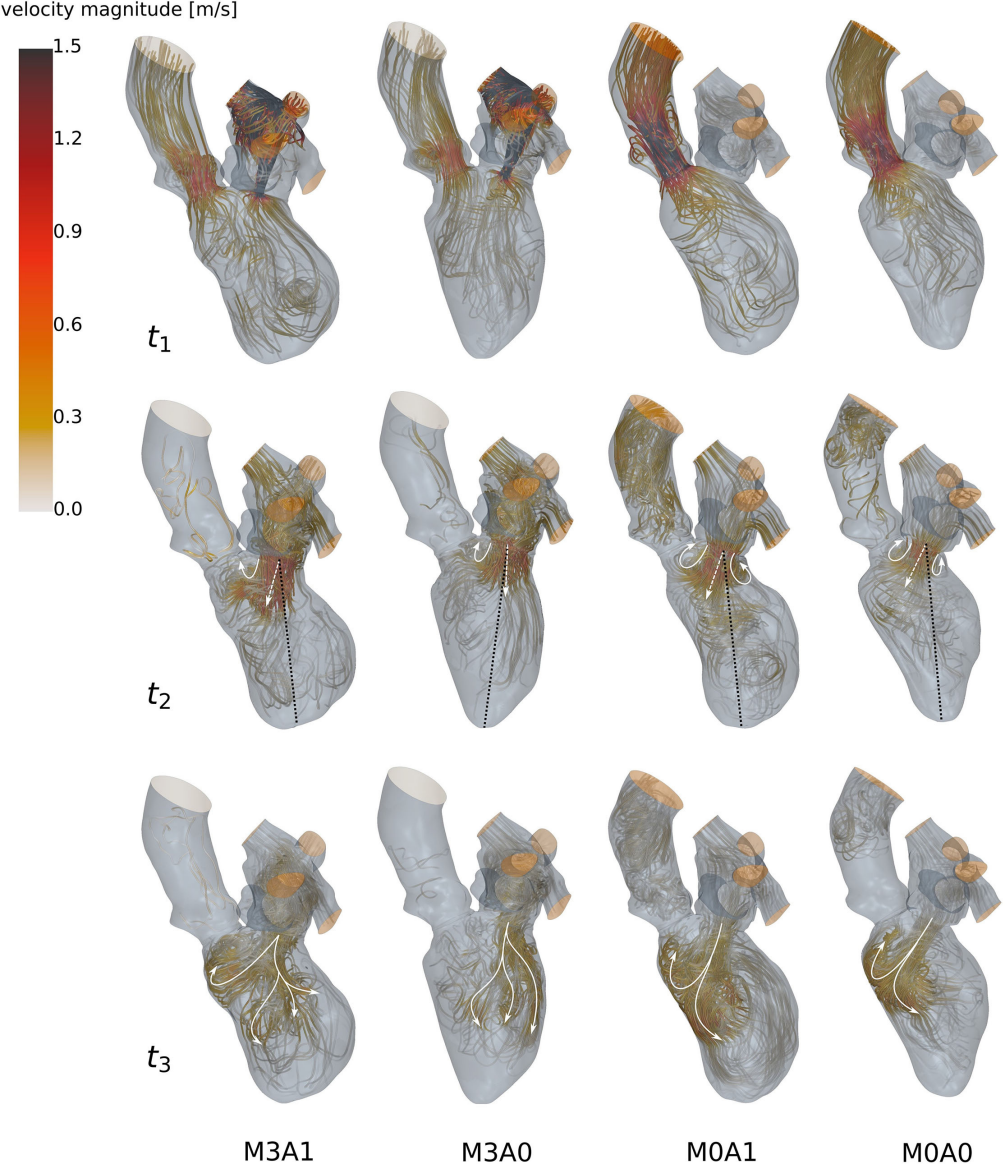
Streamlines of all four cases (left to right: M3A1, M3A0, M0A1, and M0A0) at *t*_1_: peak systole (top), *t*_2_: peak E-wave (mid), and *t*_3_: diastasis (bottom) of the seventh cycle. The white arrow, the dashed white arrow, and the dotted black line at *t*_2_ qualitatively show the vortex formation, the orientation of the E-wave jet, and the ventricular axis from MV center to apex. The white arrows at *t*_3_ schematically display the path of the E-wave jet. At low velocity regions, streamlines are made transparent and velocities above 1.5 m/s are clipped.

During systole, blood is accelerated toward the basal LV regions (Figure 6 at *t*_1_). In the non-MR cases, all blood is ejected through the LV outflow tract, whereas in the MR cases blood from the posterior-sided regions regurgitates into the LA. The regurgitating jet impinges on the upper LA wall and causes a chaotic, swirling blood motion in the LA. At the onset of diastole, a jet of blood is accelerated from the LA into the LV (Figure 6 at *t*_2_). In the MR cases, the jet transports vortex tubes, which are leftovers of the chaotic systolic flow field inside the LA caused by the regurgitation (videos M3A1-Q-criterion, M3A0-Q-criterion). Vortices are rolled up along a shear layer that forms between the resting and the inflowing blood. Whereas in the non-MR cases, a distinct ring vortex structure is visible at peak diastole, the ring structure does not entirely evolve in the MR cases (videos M3A1-Q-criterion, M3A0-Q-criterion, M0A1-Q-criterion, and M0A0-Q-criterion). Concerning the E-wave jet, its structure is being disrupted in the MR cases, such that by diastasis, it is split into several parts (Figure 6 at *t*_3_). In cases M0A1, M0A0, and partially M3A1, the jet impinges on the septal wall in mid region of the LVs in diastasis and is partially redirected toward the apex. In case M3A0, the E-wave jet moves along the posterior wall towards the apex. It is decelerated and decomposes without impinging on any wall. At late diastole (A-wave), a second jet at lower velocities rushes into all LVs. Again, vortices are rolled up along the shear layer, being weaker than the E-wave vortices. At the end of diastole, an unstructured flow field is present in all LVs.

**Figure 7:**
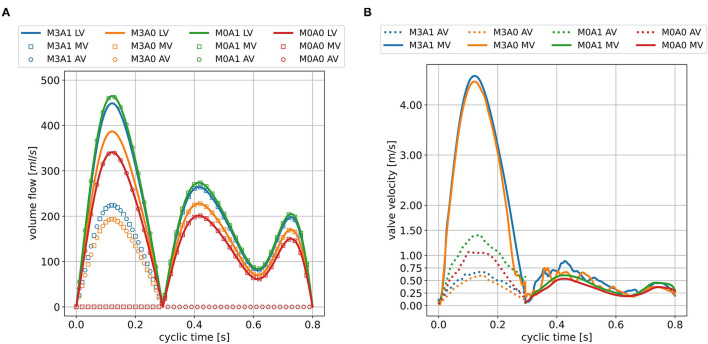
LV volume change and valve volume flow rates **(A)** and valve velocities **(B)** in the seventh cycle for all cases. The valve velocities are measured 5mm downstream of the respective valve center and are only displayed at points in time when the respective valve is open.

The peak systolic LV volume change rates range from 342 to 465 ml/s (Figure 7A). In the non-MR cases, all systolic flow leaves *via* the AV, whereas in the MR-cases, 50 % regurgitates back into the LA. The regurgitating blood exceeds velocities of 4.5 m/s in cases M3A1 and M3A0 (Table 3). The systolic AV velocities in the MR cases are lower than those in the non-MR cases (Figure 7B). In diastole, flow rates between 201 and 275 ml/s in E-wave and flow rates between 151 and 206 ml/s in A-wave, are present. The MV velocities of the non-MR cases are smooth, peaking at E-wave, and a second time at lower magnitude at A-wave. In the MR cases, the MV velocities follow the same trend, being accompanied by oscillating behavior, especially in early diastole.

### 3.2. Energetic Performance

**Figure 8:**
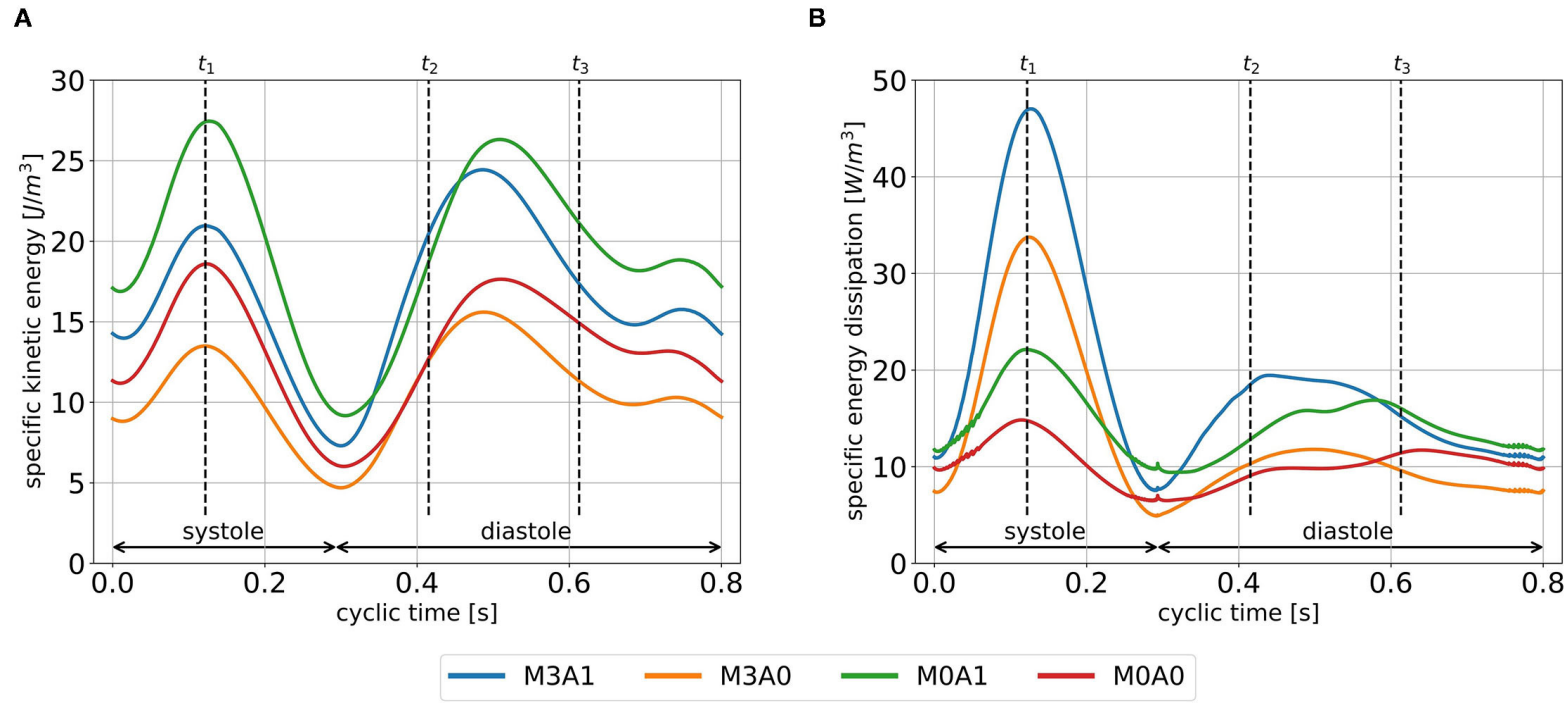
Specific kinetic energy course **(A)**, and specific energy dissipation **(B)** averaged over cycles one to eight for all cases. Time points *t*_1_, *t*_2_, and *t*_3_ mark peak systole, peak E-wave, and diastasis, respectively.

The cycle-averaged specific kinetic energy takes a qualitative similar course for all cases (Figure 8A). Local maxima are visible in peak systole, early diastole, and late diastole. In between, as the blood decelerates, local minima appear. In the non-MR cases, the global maximum is present at peak systole, whereas in the MR cases it appears at the end of early diastole when most of the vortex tubes were transported into the LV (videos M3A1-Q-criterion, M3A0-Q-criterion). The cycle-averaged specific kinetic energy magnitude is highest for cases M3A1 and M0A1, which are characterized by larger SVs.

Figure 8B displays the cycle-averaged specific energy dissipation. In the MR cases, higher specific energy dissipation is observable in systole. When comparing case M3A1 to M0A1 and case M3A0 to M0A0, where the SVs are very similar, the specific energy dissipation in peak systole is more than two times as high in the MR cases. In diastole, the maximum specific energy dissipation in the MR cases appears during early diastole, whereas in the non-MR cases, it is visible in diastasis. By taking the volume integral of the energy dissipation, a power loss can be calculated (30). The application of a time integral over the cardiac cycle can then reveal the cyclic power loss. Computing that cyclic power loss per case, it is revealed, that over a cardiac cycle, the MR cases dissipate approximately the same amount of energy as they have left at the end of that cycle, whereas the non-MR cases only dissipate around 60 % (cyclic power loss/end-diastolic kinetic energy in M3A1: 92.9; M3A0: 100.5; M0A1: 58.5; and M0A0: 64.1 %).

### 3.3. Intraventricular Washout

**Figure 9:**
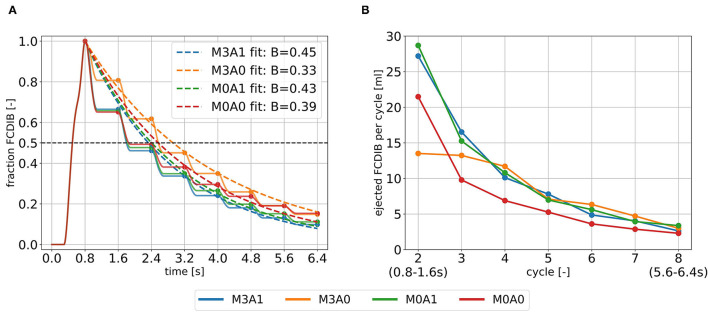
Intraventricular washout of FCDIB until the end of cycle eight **(A)** and the respective ejected fractions of FCDIB per cycle **(B)**. To perform the exponential fit, the FCDIB fractions at the end of each cycle were used [marked as dots in **(A)**].

The blood in which the passive scalar was placed to track the fluid motion, the FCDIB, enters the LV in diastole of the first cycle and is ejected in the subsequent cycles (Figure 9A). The ejection evolutes in a staircase-like manner with reducing stair sizes. Characteristic washout measures are displayed in Table 4. The 99 % washout is computed *via* the exponential fit function in Equation (8). The R^2^ values (quality criterion for regression models) for the exponential fits are above 97 % for all cases.

**Table 4:** Intraventricular washout measures of the four representative cases.

**Parameters**	**M3A1**	**M3A0**	**M0A1**	**M0A0**
direct flow rate (%)	33.5	19.3	34.2	34.9
direct flow rate (ml)	27.2	13.5	28.7	21.5
half-life FCDIB (s)	1.80	2.57	1.82	1.85
99% washout FCDIB (s)	10.95	14.88	11.46	12.52

*The half-life marks the time when 50% of FCDIB are ejected. The 99% washout marks the time when 99 % of FCDIB are ejected and is computed via an exponential fit function (Equation 8). FCDIB, first cycle diastolic inflowing blood*.

The ejected fractions of FCDIB per cycle are displayed in Figure 9B. Cases M3A1, M0A1, and M0A0 behave similar in a decreasing exponential shape, whereas the trend is linear in case M3A0. As a result, similar fractions of FCDIB inside the LVs are present at the end of cycle eight at 6.4 s (Figure 9A). A slightly worse washout is observed in the non-aneurysmatic cases M3A0 and M0A0. At the end of cycle eight, approximately 5 % more FCDIB are still present in the LVs in the non-aneurysmatic cases. They furthermore incorporate lower medium velocities in the apex region during systole and diastole and a slightly increased FCDIB accumulation in that region at the end of cycle eight (see Supplementary Material).

**Figure 10:**
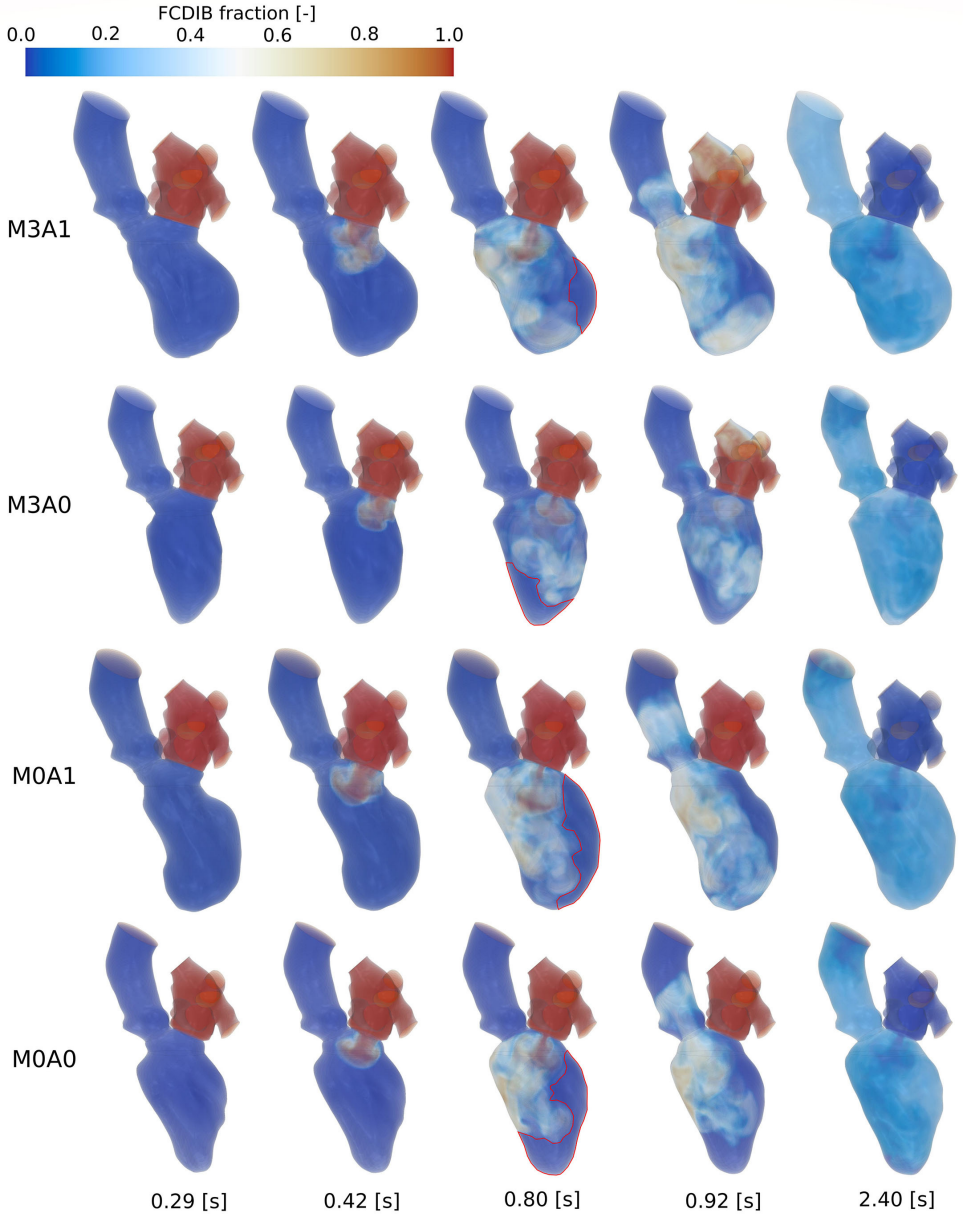
FCDIB for all cases (top to bottom: M3A1, M3A0, M0A1, M0A0) from left to right at end systole of the first cycle (0.29 s), peak E-wave of the first cycle (0.42 s), end diastole of the first cycle (0.8 s), peak systole of the second cycle (0.92 s), and the end of the third cycle (2.4 s). Red contours at end diastole of the first cycle mark regions with poor washout, which are associated with a higher risk of thrombus formation.

Figure 10 shows the FCDIB over several points in time of the first three cycles. At end systole of the first cycle (0.29 s), the entire LA is filled with the passive scalar. In peak E-wave of the first cycle (0.42 s), the FCDIB jet rushes into the LV. In the non-MR cases, the jet is coherent, whereas in the MR cases, it already starts disintegrating. By the end of the first cycle (0.8 s), regions the FCDIB did not reach are visible (see red marked contours in Figure 10). These regions differ from case to case (M3A1: concentrated lateral region; M3A0: septal and apical regions; M0A1: elongated lateral region; M0A0: lateral and apical regions) and are partially still visible in peak systole of the second cycle (0.92 s). It furthermore becomes visible, that the non-MR cases eject a larger fraction of FCDIB into the AO than the MR cases, in which some of the FCDIB regurgitates into the LA. At the end of the third cycle (2.4 s), the remaining FCDIB is distributed mostly homogeneous inside the LVs.

## 4. Discussion

In this study, we propose a moderate complexity, generally applicable, and quick to preprocess modeling approach to calculate intraventricular hemodynamics with image-based CFD. The ventricular movement is derived from the segmented end-systolic and end-diastolic geometries and prescribed in an arbitrary Lagrangian-Eulerian formulation at the boundaries. The valves are modeled as 2D orifices, whereat the MV is represented by a projected orifice shape. LA and AO geometries are attached at the annuli and physiological pressure curves, respective mass flow rates are posed as inflow and outflow BCs. This methodology requires only end-diastolic and end-systolic geometries and is currently focused on CCT, which typically has a rather low temporal resolution due to the desired minimization of radiation dose and technical limitations. However, it is applicable in the same manner to MRI and echocardiographic data. To demonstrate the technical and clinical feasibility, the methodology was applied to four cases, each representing a subcohort of a pathological state of heart failure patients after myocardial infarction.

### 4.1. Intraventricular Hemodynamics

We evaluated the intraventricular hemodynamics of the four representative cases in terms of large-scale flow patterns, energetic behavior, and washout. The most noticeable observations were made in the MR cases. In these, no clear ring vortex structure evolves during the diastolic inflow. The E-wave jet is disrupted by the transported vortex tubes and the accompanying swirling motion supposedly prevents a distinct shear layer development, leading to fragmentary vortex formation. In the study of Le and Sotiropoulos (28), similar observations were made for wall-extracted secondary vortex tubes that cause instabilities in the circumferential direction of the vortex ring. Subsequently, the E-wave jet is decomposed in the temporal course of diastole in the MR cases, resulting in an unstructured, vortical flow. In terms of energetic behavior, the MR cases have their global specific kinetic energy maximum in early diastole, whereas it appears in systole in the non-MR cases. These observations correlate well with the findings of Al-Wakeel et al. (15), where MR patients were examined with 4D flow MRI. Therein, an unstructured diastolic LV flow field with several smaller vortex structures and an increased early diastolic kinetic energy state in MR patients that reduces after MV surgery were seen. The increased early diastolic kinetic energy state might be explained by the vortex tubes flowing from the LA into the LV during early diastole, storing kinetic energy. Regarding the temporal course of specific energy dissipation in diastole, the maximum values in the MR cases appear in early diastole, whereas it peaks in diastasis in the non-MR cases. Possible explanations can be found in the early diastolic decaying vortex structures and the jet impingement on the septal wall in diastasis. It furthermore strikes out, that the MR cases reveal a significantly increased specific energy dissipation in systole. To make estimates about the overall degree of abnormality of the energetic behavior in these pathological cases, a comparison to energetic states of a healthy LV analyzed in the same modeling approach seems promising.

In addition to the findings when comparing MR to non-MR cases, two further interesting aspects are to highlight. First concerns the intraventricular washout. Our results suggest that considering several cycles might provide a more detailed view of the washout process. When tracking the FCDIB over several cycles, it strikes out that case M3A0 has a significantly lower direct flow rate, but the fraction of FCDIB inside the LV catches up to the other cases by the end of the eighth cycle. We extrapolated the washout *via* a straightforward exponential fit function. The resulting fits overestimate FCDIB in the first cycles and underestimate FCDIB in the later ones or vice versa (Figure 9A). Such a discrepancy is likely to be transferred onto the extrapolation. However, this deviation can potentially be overcome by computing more cycles or applying enhanced fit functions (e.g., the sum of two time-weighted exponential functions), which might be interesting as a 99 % washout time could serve as a valuable metric for clinicians to quantify blood residence times, that CFD can provide.

In terms of tracking the FCDIB, it is also noticeable that the aneurysmatic LVs are characterized by a better washout. At first sight, this seems contra-intuitive. Yet, differences are small, and it must be taken into account that the non-aneurysmatic cases have higher EFs and SVs. Furthermore, all LVs suffered a myocardial infarction and are dilated with reduced EFs, thus being characterized by a pathological state.

The second aspect relates to the regions the FCDIB did not reach at the end of the first cycle, which are partially still present in the systole of the second cycle (Figure 10). These regions can be associated with a worsened washout. In cases M3A1, M0A1, and M0A0, mainly lateral regions are affected. In these cases, the E-wave jets are oriented away from the lateral regions toward the septal wall (Figure 6 at *t*_2_), suggesting a correlation between E-wave jet orientation and washout. Based on this, the question arises as to what determines the inflow orientation. We identify two potentially interesting relationships in this study. First concerns the deformation. In cases M3A1 and M0A1, the strongest deformation appears in septal and inferior regions, e.g., segments 5 and 6 (Figure 2A). As these regions expand strongest in diastole, they are prone to have a suction effect on the inflow. The second relates to the angle β between the normal on the MV plane and a vector connecting the MV center to the apex at LVEDV. This angle lies between 30° and 31° for cases M3A1, M0A1, and M0A0, whereas it is only 14.3° for case M3A0. The large angles indicate an inflow being oriented toward the septal walls. A link between the inflow angle and an abnormal LV flow field was also observed in the study of Witschey et al. (14). Such a causal chain from local deformation and angle β *via* the inflow orientation toward the intraventricular washout would be insofar interesting, as it links purely geometric metrics with the washout. This would allow clinical assessments without the need for actual flow measurements or CFD. However, for further investigations of such a relationship, a larger database needs to be considered and the influence of neglecting the 3D valve topology must be reviewed in this context.

It is to say, that this pilot study is based on a small number of investigated clinical cases and is thus not suited for statistical analysis and generalized statements of findings regarding intracardiac flow feature differences between the subcohorts. In general, the question arises whether intracardiac flow measures may, besides the usage to quantify and analyze the LV flow field, serve as biomarkers for an early prediction of LV diseases. This topic is currently discussed (9, 24, 61). As this study only covers snapshots of the LV states, the progression of the diseases cannot be assessed. However, we are aiming to make use of the low pre-processing time of the presented methodology to investigate the entire available LV cohort and search for patterns in different manifestations of the diseases, which might provide further insights.

### 4.2. Methodology

The proposed computational framework is set up at moderate complexity to obtain reasonable results for the intraventricular hemodynamics based on CCT in clinically realizable time frames. A modeling approach of this sort comes at the price of simplifications, which must be critically reviewed. In terms of the geometric level of detail, the negligence of papillary muscles and trabeculae must be noted. Lantz et al. (62) and Vedula et al. (63) simulated LV flow dynamics based on high-resolution CCT, including papillary muscles and trabeculae, and compared it to the same LVs excluding these features. In both publications, local flow differences become visible and, e.g., stagnant flow in the papillary muscle region (62) and an increased viscous dissipation rate in early diastole (63) are observed in the detailed LVs. Yet, for example, the specific kinetic energy course did not extensively differ between the cases in the study of Vedula et al. (63). Including papillary muscles and trabeculae and investigating their influence is planned in upcoming model development steps.

Validation of the 2D valves is currently ongoing. We incorporated 2D generic valves as adequate 3D patient-specific valves could not be obtained for all cases from the available CCT data. We followed the approaches by Schenkel et al. (25) and Daub et al. (51) that implemented the 2D-planar valve model in previous studies. As the valves are simplified, not the entire MV apparatus, e.g. chordae tendineae, is modeled. The inclusion of the chordae into the MV model could however be necessary for biomechanical studies (64) or realization of a 3D MV movement using fully-coupled FSI (55). Yet, it is to note, that Morud et al. (65) found a negligible impact of chordae on the systolic intraventricular flow. Including chordae can furthermore be associated with time-consuming efforts in pre-processing. In case our ongoing studies indicate non-negligible differences between the valve models, the model will be adapted.

At the annuli, we attached available non-patient-specific high-quality segmentations of LA and AO. The aortic flow field and the patient-specific AO geometry are not of particular interest in this study. Concerning the LA, Mihalef et al. (66) compared vortex structures of the same configuration with and without including the LA. In their study, they observed that the main differences in terms of vortical flow come from the transported vorticity from the LA into the LV. Schenkel et al. (25) demonstrated a dependency of the LV flow field on the LA geometry by incorporating two different generic LA structures. The extent to which the LA geometry must be patient-specific and whether its motion must be represented to adequately model the LA-LV coupling remains to be clarified. The same applies to a representation of the arterial system at the AO outlet and the concomitant ventriculo-arterial coupling.

The ventricular motion is based on the end-diastolic and end-systolic geometries under the simplifying assumption of a longitudinal homogeneous contraction and negligence of the torsional motion. Including intermediate states would be desirable but was not adequately possible due to the temporal resolution of the CCT data. Yet, the imposed ventricular deformation is able to follow the specified volume curve at high precision. Additionally, the ventricular movement, with the simulation starting at the onset of systole at LVEDV, is able to closely match the respective segmented end-systolic geometry. Under these considerations, the prescribed motion can be verified. The mesh quality preservation and the results of the mesh independence study furthermore confirm an adequate computational mesh. It is left to point out, that in this pilot study we focused on establishing the workflow and performing a proof of concept. Further model enhancements and detail inclusion will be the subject of future developments.

In this study, we focused on the evaluation of the intraventricular flow field and investigated several hemodynamic parameters. The model allows the examination of further quantities like wall-shear-stress or blood residence times, such that in total, a broad range of hemodynamic markers is covered. Yet, to be able to investigate, for example, the pressure-volume loop, valve opening and closing times or tissue stresses and strains, complex modeling approaches that include tissue mechanics and potentially electrophysiology are required. Such modeling approaches are pursued, e.g., by Karabelas et al. (67), who introduced an electro-mechano-fluidic model or Mao et al. (55), who developed a fully-coupled FSI model including both valves. Despite having the potential to describe all aspects of cardiac physiology, these multi-physics models tend to have high requirements on data quality, introduce additional modeling uncertainties, and require additional resources for pre-processing, model parameterization, and computation, which might, however, impact clinical usability.

In comparison to *in vivo* measurements of hemodynamic quantities *via* flow MRI (16) or echocardiography (13, 23), the proposed computational framework introduces uncertainties due to model simplifications, especially in terms of anatomical shapes and resolution of fine structures (e.g., chordae tendineae, trabeculae, and valve leaflets). On the other hand, it allows for the computation of hemodynamics at considerably higher spatio-temporal resolution and enables a direct calculation of pressure fields and intraventricular washout over various cardiac cycles, whereas image-based *in vivo* measurements are unable to directly measure pressure fields. To make use of these advantages of the proposed CCT-based CFD framework, a validation with, e.g., 4D flow MRI is required to quantify the impact of the mentioned simplifications. Such a validation is planned in the near future, taking into consideration various hemodynamic parameters which are associated with pathological LVs.

### 4.3. Clinical Feasibility

The proposed computational framework is motivated by developing image-based CFD models toward a clinical feasibility. Beside data availability and model quality, the required working hours, computing time, and computational expenses affect the prospective clinical feasibility. Currently, pre-processing by an experienced user takes 6-8 h to get from medical images to a simulation setup, which is ready for computation. Further automation in the segmentation and pre-processing procedures offers the potential to reduce these working hours as well as to minimize possible impacts of operator-dependent manual interactions. Inter-user variability is known in the context of segmentation or surface preparation of geometric models, influencing the resulting cardiovascular structures and therewith the intraventricular flow (68, 69). The computation time per cycle at the specified CPU number results in computational costs of approximately 150 to 200 € per cycle at the North-German Supercomputing Alliance. In this study, we primarily focused on computational accuracy. An optimization toward low computational costs can significantly reduce these expenses.

Besides the costs per cycle, the investigated number of cycles is crucial for overall cost estimation. In this work, we computed five cycles in advance before assuming a swung in the state and considering the results for the evaluation. To review whether less than five cycles would be sufficient to receive a swung in state, we computed the mean cyclic energy for each of these in advance compute five cycles and compared it to the mean cyclic energy of the consecutive eight cycles that were used for the evaluation. The relative changes were 34.6–38.3% for the first cycle and ranged from 0.6–9.7% for cycles two to five. From an energetic perspective, it thus seems justifiable to only compute one cycle in advance for LVs at comparable volumes and EFs.

In the evaluated eight cycles, the specific kinetic energy, specific energy dissipation, and the averaged valve velocities and transvalvular pressure drops show sufficiently small cyclic deviations to be evaluated in solely one cycle. The large-scale flow patterns behave qualitatively alike over all cycles, considering cyclic variations, which are also emphasized by Chnafa et al. (26). In terms of intraventricular washout, our results indicate that considering several cycles might provide a more detailed view of the washout process.

Taking these considerations into account, the here proposed computational framework yields orders of magnitude for pre-processing, solving, and post-processing, that are within range of clinical feasibility. Furthermore, there is still potential to minimize the need for manual interaction as well as pre-processing and computational times.

## 5. Conclusion

In this study, we propose a CCT-based CFD framework to compute intraventricular hemodynamics on a patient-specific level to complement the informative value of CCT. The computational framework enables pre-processing and computational times, respective expenses at an order of magnitude that is within reach of clinical feasibility. The technical feasibility was verified by means of four cases, each representing a subcohort with a respective pathology. Where possible, the plausibility of the results was confirmed by comparison with other publications. Comparing the four cases, the most noticeable observations were made in the LVs with MR. Developments toward the inclusion of geometric details and patient-specific valve geometries have the potential to provide further insights into the disease. We conclude the proposed computational framework to have a good potential of becoming clinically usable as it represents a fair balance between model accuracy and overall expenses. Further model refinement, workflow automation, computation acceleration, and a validation with 4D flow MRI are the next steps toward enabling a clinical translation and making image-based CFD usable to support clinical diagnosis and treatment planning in everyday routine.

## Data Availability Statement

The data sets (geometries and applied boundary conditions) presented in this study are provided as open data in an online repository. The name of the repository and accession number can be found at: https://doi.org/10.6084/m9.figshare.16964929.

## Ethics Statement

The studies involving human participants were reviewed and approved by the Ethics Committee-Charité Universitätsmedizin Berlin (EA2/177/20). The study was performed according to the principles of the Declaration of Helsinki.

## Author Contributions

LG, NS, CK, and TK: conceptualization. LO, AS, NS, and CK: data curation. LO, LT, KV, JB, and LG: formal analysis and methodology. LG and TK: funding acquisition and supervision. LO and AS: visualization. LO, NS, CK, and LG: investigation. LO, KV, and LG: writing original draft. All authors review and editing. All authors contributed to the article and approved the submitted version.

## Funding

This study was funded by the Einstein Center Digital Future and by the DFG grants for the project Nr. 465178743 in frames of the SPP2311.

## Conflict of Interest

The authors declare that the research was conducted in the absence of any commercial or financial relationships that could be construed as a potential conflict of interest.

## Publisher's Note

All claims expressed in this article are solely those of the authors and do not necessarily represent those of their affiliated organizations, or those of the publisher, the editors and the reviewers. Any product that may be evaluated in this article, or claim that may be made by its manufacturer, is not guaranteed or endorsed by the publisher.

## Supplementary Material

### Supplemental Data

A mesh independence study and a regional ventricular analysis as well as the videos of the FCDIB (M3A1- FCDIB, M3A0-FCDIB, M0A1-FCDIB, and M0A0-FCDIB) and the Q-criterion (M3A1-Q-criterion, M3A0-Q-criterion, M0A1-Q-criterion, and M0A0-Q-criterion) are provided as supplemental data.
