# Supplementary material for: CT-Based Simulation of Left Ventricular Hemodynamics: A Pilot Study in Mitral Regurgitation and Left Ventricle Aneurysm Patients
Source: Front Cardiovasc Med. 2022 Mar 22;9:828556. doi: 10.3389/fcvm.2022.828556 (PMC8980692; doi:10.3389/fcvm.2022.828556)
Supplement: Supplementary file 1 [file Data_Sheet_1.PDF]

Supplementary Material

1 MESH INDEPENDENCE STUDY

To investigate mesh convergence, the velocity magnitudes along three line probes (see Figure S1A) are evaluated for different base sizes exemplary for case M0A0. The velocities are measured at peak systole and peak E-wave for base sizes of 2 mm, 1.5 mm, 1.25 mm, 1 mm, 0.9 mm, and 0.8 mm. In valve regions, the base size is refined to 25 %. To minimize computational expenses, the line probes are evaluated in the first cycle after a swung in state was reached. Figures S1B and C display the relative L1 and relative L2 norm referring to the velocity magnitudes of the finest base size of 0.8 mm.

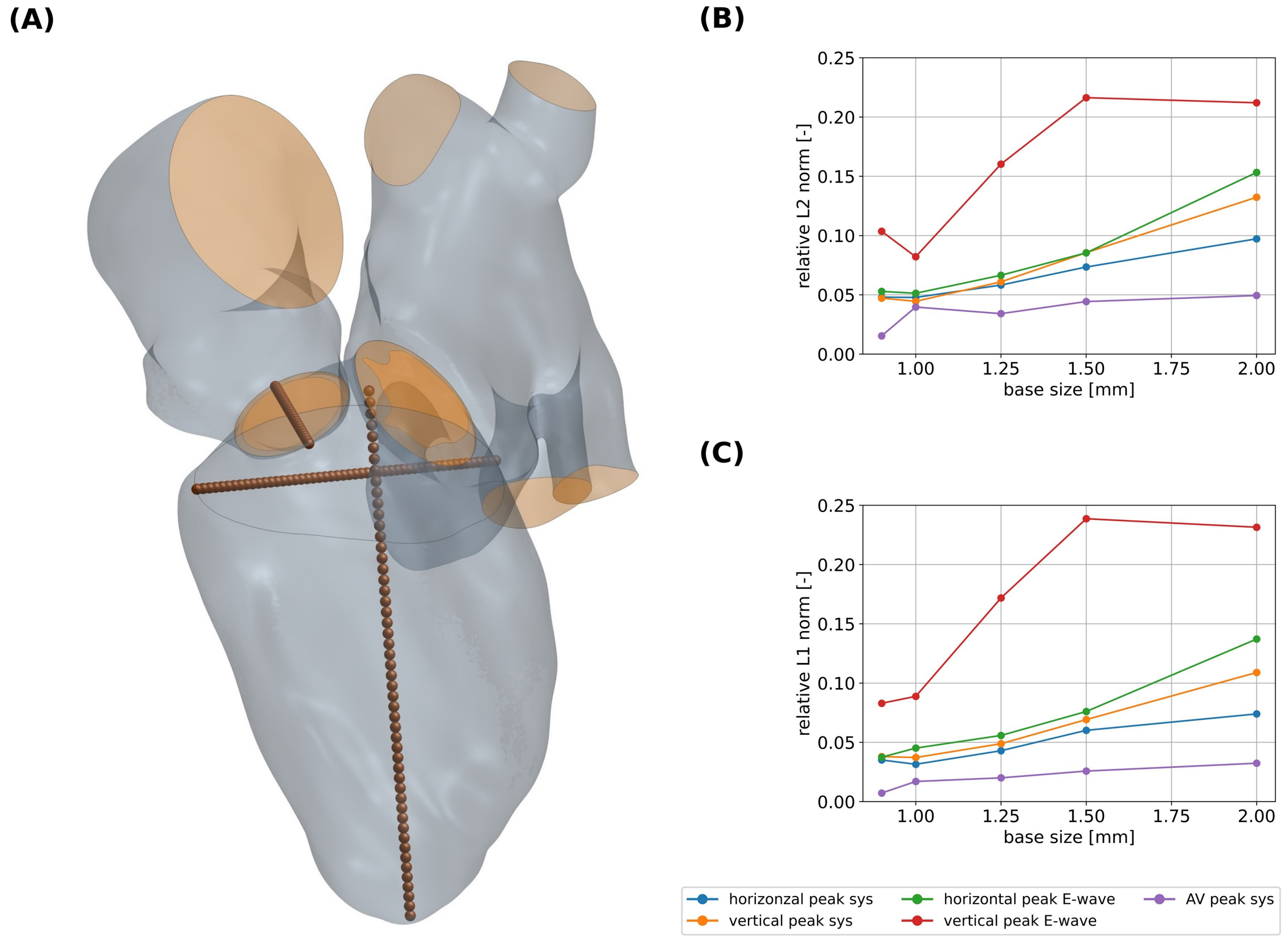

Figure S1. Lines probes used for the mesh independence study **(A)** and resulting relative L1 **(B)** and relative L2 **(C)** errors for the velocity magnitude for different base sizes. The vertical line probe expands from base to apex along the ventricular axis and is composed of 50 points. The horizontal line probe expands in basal region from anterior to posterior wall and is also composed of 50 points. The aortic valve (AV) line probe is positioned along the long axis of the aortic annulus and consists of 25 points.

Figure S2 displays the velocity magnitudes along the respective line probes for base sizes of 2 mm, 1.5 mm, 1.0 mm, and 0.8 mm.

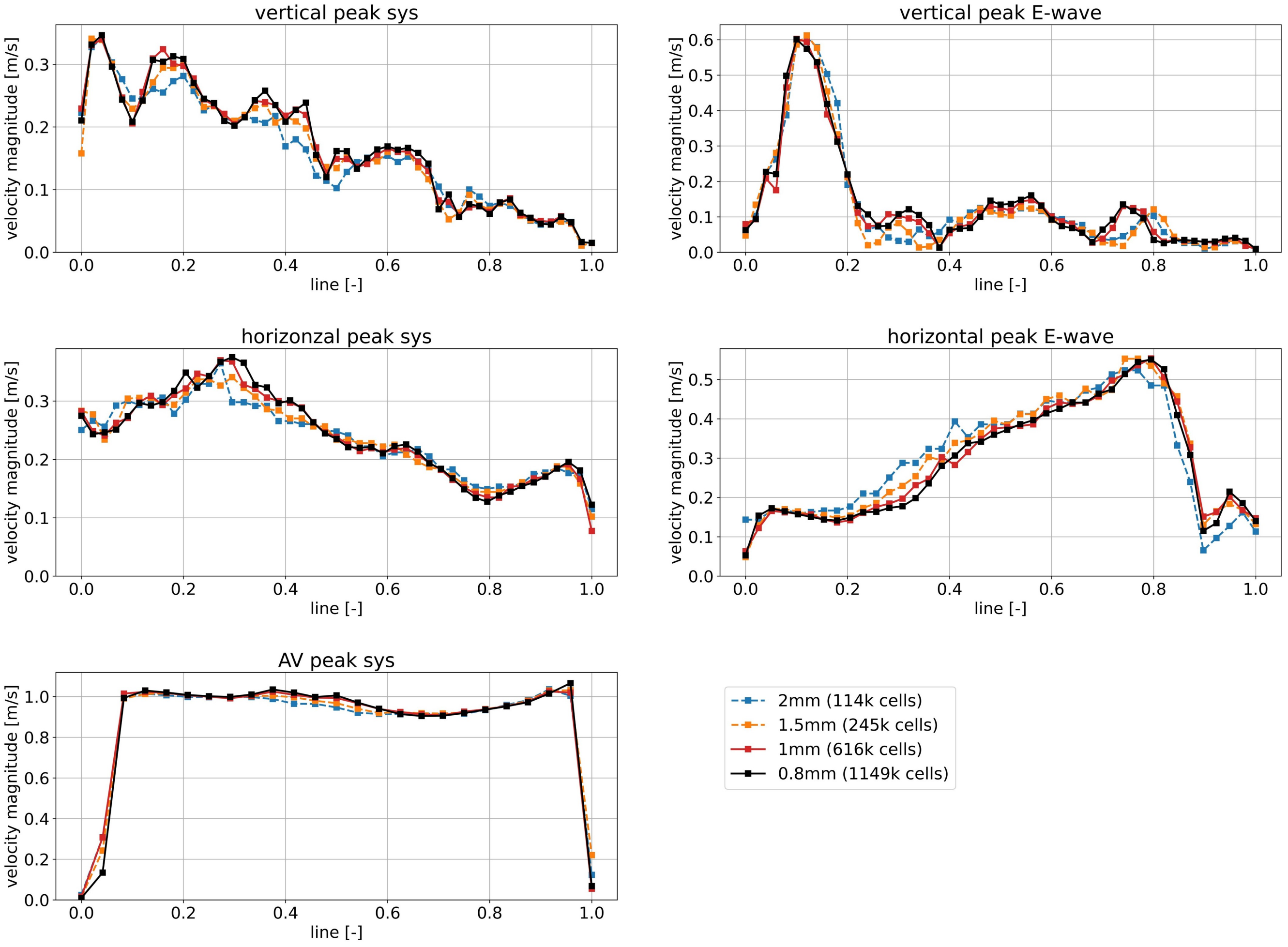

Figure S2. Velocity magnitudes along the different line probes at the respective time points as specified in the graph titles.

A base size of 1 mm with a mesh refinement to 0.25 mm in valve regions leads to the mesh characteristics as displayed in Table S1. Figure S3 exemplifies the resulting mesh volumes for case M0A0.

Table S1. Mesh characteristics of the four representative cases.

Parameters	M3A1	M3A0	M0A1	M0A0
total cell count (-)	694693	707375	670047	616357
cell count LV (-)	367809	391106	363930	321170
cell count LA (-)	193134	186653	180950	170506
cell count AO (-)	133750	129616	125167	124681
total number of vertices (-)	4235985	4319341	4101038	3767900
average cell volume (mm^3)	1.24	1.26	1.28	1.25

LV: left ventricle, LA: left atrium, AO: aorta.

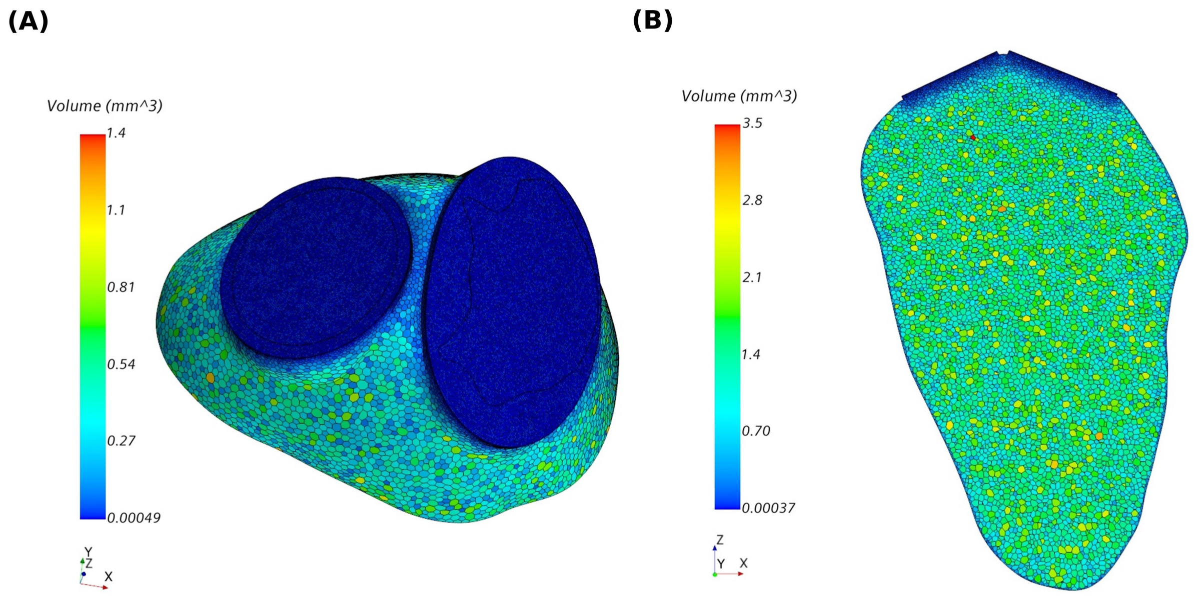

Figure S3. Mesh volumes at the boundaries of the left ventricle (LV) in valve region **(A)** and in a half cut through the LV **(B)** for case M0A0. The cutting plane in **(B)** is defined via the valve centers and the apex.

2 REGIONAL VENTRICULAR ANALYSIS

To quantify regional differences in the LVs, they are separated into 17 regions of interest (ROIs) and analyzed in terms of velocity magnitude and first cycle diastolic inflowing blood (FCDIB). The ROIs are qualitatively arranged as the segments in the 17-segment model. To clearly distinct the ROIs during the ventricular movement, they are defined as follows. The average of the diastolic and systolic LV centerline is computed. A mesh cell is then assigned to a ROI based on its longitudinal distance to the base and its angle to that centerline in circumferential direction. Figure S4 displays the splits for the end-diastolic and end-systolic geometries of case M3A1.

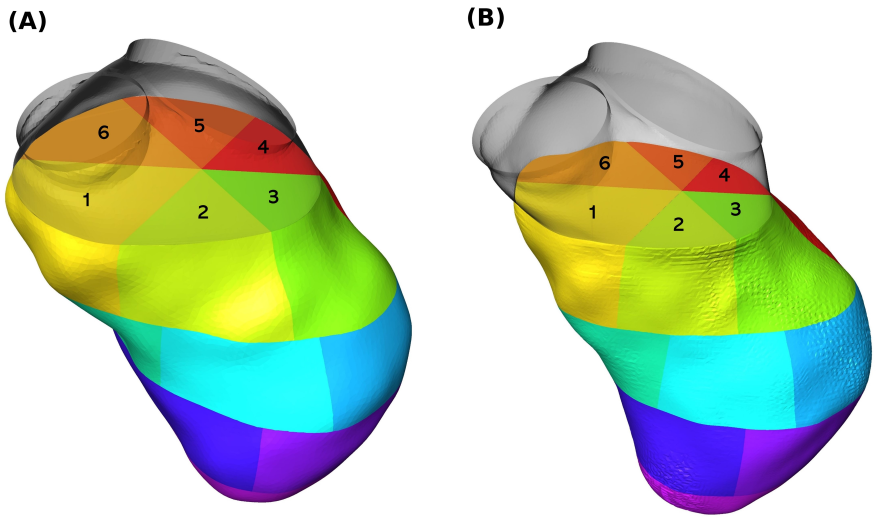

Figure S4. ROIs of the end-diastolic **(A)** and end-systolic **(B)** geometries of case M3A1 with marked ROIs 1 - 6.

The average diastolic and systolic velocities decrease from base to apex with minimum velocities being present in apex region (ROI 17) in all cases (Figure S5A and B). Slightly lower velocities in apex region

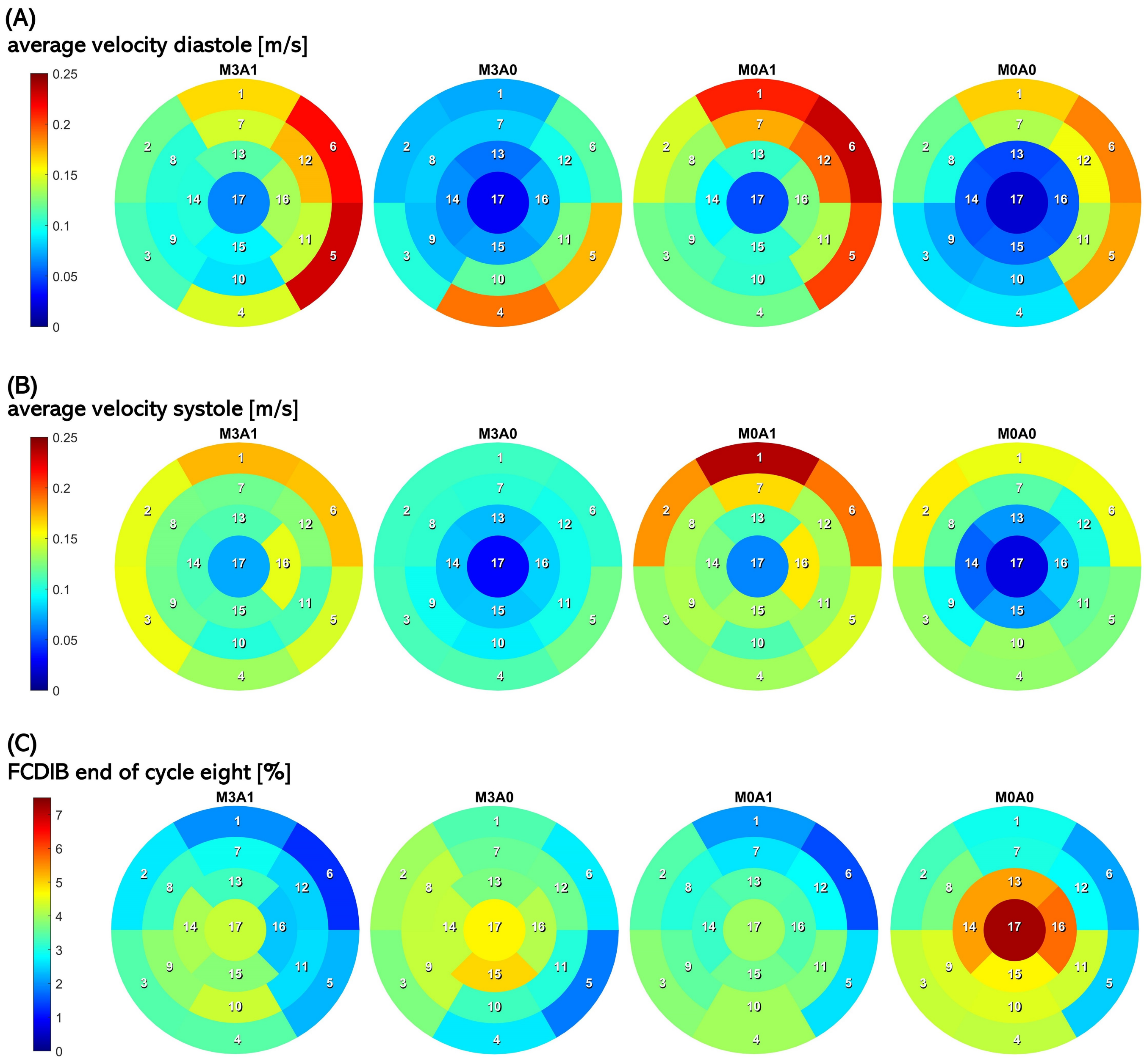

Figure S5. 17-segment visualizations of the ROIs of the representative cases for the average diastolic velocity **(A)**, the average systolic velocity **(B)** and the FCDIB at the end of cycle eight **(C)**. The velocities are evaluated every 0.05 s and are averaged over all eight cycles.

are observable in the non-aneurysmatic cases compared to the aneurysmatic cases. In these regions, also the largest fractions of FCDIB are present at the end of cycle eight (Figure S5C). Otherwise, the FCDIB is distributed mostly homogeneous over the LVs. The average diastolic velocities also illustrate the E-wave jets to be directed towards the septal wall for cases M3A1, M0A1 and M0A0, respectively along the posterior wall for case M3A0 by means of increased velocities in these regions. In systole, the highest

average velocities in the cases without mitral regurgitation (MR) are visible in ROIs 1, 2, and 6, below the AV. In the MR cases, the average systolic velocities are distributed more uniformly in basal region.
