# Supplementary figures and images for: CT-Based Simulation of Left Ventricular Hemodynamics: A Pilot Study in Mitral Regurgitation and Left Ventricle Aneurysm Patients
Source: Front Cardiovasc Med. 2022 Mar 22;9:828556. doi: 10.3389/fcvm.2022.828556 (PMC8980692; doi:10.3389/fcvm.2022.828556)

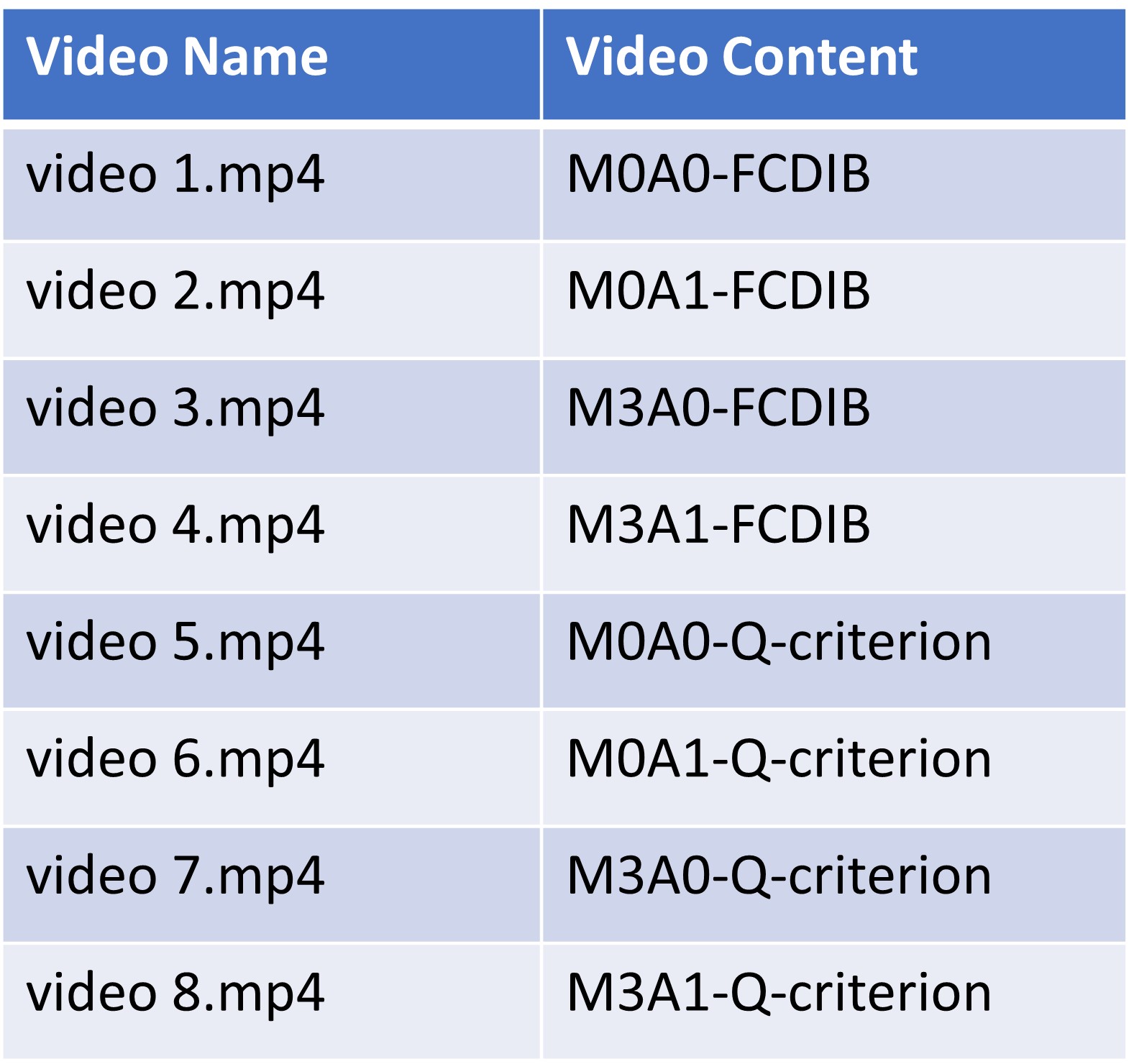

Supplement: Supplementary file 2 [file Image_1.jpg]
